# Discovering candidate imprinted genes and imprinting control regions in the human genome

**DOI:** 10.1186/s12864-020-6688-8

**Published:** 2020-05-31

**Authors:** Minou Bina

**Affiliations:** Department of Chemistry, Purdue University, 560 Oval Dr., West Lafayette, IN 47907 USA

**Keywords:** Gene regulation, Genetics, Developmental, Genomic imprinting, Mammals, MLL, Syndrome, ZFP57

## Abstract

**Background:**

Genomic imprinting is a process thereby a subset of genes is expressed in a parent-of-origin specific manner. This evolutionary novelty is restricted to mammals and controlled by genomic DNA segments known as Imprinting Control Regions (ICRs) and germline Differentially Methylated Regions (gDMRs). Previously, I showed that in the mouse genome, the fully characterized ICRs/gDMRs often includes clusters of 2 or more of a set of composite-DNA-elements known as ZFBS-morph overlaps.

**Results:**

Because of the importance of the ICRs to regulating parent-of-origin specific gene expression, I developed a genome-wide strategy for predicting their positions in the human genome. My strategy consists of creating plots to display the density of ZFBS-morph overlaps along the entire chromosomal DNA sequences. In initial evaluations, I found that peaks in these plots pinpointed several of the known ICRs/gDMRs along the DNA in chromosomal bands. I deduced that in density-plots, robust peaks corresponded to actual or candidate ICRs in the DNA. By locating the genes in the vicinity of candidate ICRs, I could discover potential imprinting genes. Additionally, my assessments revealed a connection between several of the potential imprinted genes and human developmental anomalies. Examples include Leber congenital amaurosis 11, Coffin-Siris syndrome, progressive myoclonic epilepsy-10, microcephalic osteodysplastic primordial dwarfism type II, and microphthalmia, cleft lip and palate, and agenesis of the corpus callosum.

**Conclusion:**

With plots displaying the density of ZFBS-morph overlaps, researchers could locate candidate ICRs and imprinted genes. Since the datafiles are available for download and display at the UCSC genome browser, it is possible to examine the plots in the context of Single nucleotide polymorphisms (SNPs) to design experiments to discover novel ICRs and imprinted genes in the human genome.

## Background

Imprinted genes play key roles in fetal development and in postnatal processes including behavior, sleep, feeding, maintenance of body temperature, metabolic regulation, and stem cell maintenance and renewal [1]. The imprinting mechanism is relatively complex and involves orchestrated actions of several enzymes and proteins [2, 3]. In that process, key players include ZFP57 and a complex consisting of DNMT3A and DNMT3L. This complex methylates DNA processively on a variety of CpG-rich substrates including the promoters of human genes encompassed by CpG islands [4]. Because of the importance of the ICRs to parent-of-origin specific gene expression, it is necessary to develop strategies for their localization in mammalian genomic DNA. Towards that goal, a previous study discovered that ZFP57 interacted with a double-stranded, CpG-methylated, hexanucleotide (TGCCGC). This interaction was essential to the recognition of ICRs by ZFP57 to maintain allele-specific gene expression [5]. Since CpG containing sequences are infrequent in animal DNA [6], the instances of finding ZFBS in mammalian DNA is by far less than those observed for AT-rich hexanucleotides. Nonetheless, TGCCGC is relatively short. Therefore, it occurs often in genomic DNA [7]. Consequently, random occurrences of the hexameric site would obscure detection of the functional ZFP57 sites within the ICRs/gDMRs dispersed along relatively long genomic DNA sections.

Previously, I addressed that problem by extending the length of the canonical ZFP57 binding site to include additional nucleotides [7]. This strategy eliminated a significant fraction of randomly occurring ZFP57 sites in mouse genomic DNA and led to the discovery of the ZFBS-Morph overlaps [7, 8]. These overlaps define composite-DNA-elements consisting of the hexameric ZFP57 binding site overlapping a subset of the MLL1 morphemes [7, 8]. These morphemes correspond to the smallest ‘words’ in DNA that selectively bind the MT-domain in MLL1 [9]. The MT domain is present in both MLL1 and MLL2. In DNA binding assays, this domain interacted selectively with nonmethylated CpG-rich sequences [10, 11]. Thus, within the ICRs, the ZFBS-Morph overlaps may play a dual and antagonistic role. Binding of MLL1 and MLL2 to ICRs protecting the DNA from methylation. Binding of ZPF57 to the modified DNA maintaining allele-specific expression [5, 7, 10–12].

Since closely-spaced ZFBS-Morph overlaps impart contextual specificity to ICRs, their localization could help with pinpointing the genomic positions of the ICRs that are currently unknown. Towards this goal, I describe a Bioinformatics strategy. This strategy involved creating plots to view the positions of clusters of 2 or more ZFBS-Morph overlaps along chromosomal DNA. In these plots, peaks appear within a sliding window consisting of 850-bases. By displaying the plots at the UCSC genome browser, I could locate the peaks corresponding to several of the well-known ICRs/gDMRs. I also could examine the human genes in the context of short clinical variants and results of genome wide association studies. Here, I give an overview of how with density-plots, I could discover potential imprinted genes. I found that several of these genes were associated with disease-states and developmental anomalies know as syndromes. Examples include association of *IMPDH1* with Leber congenital amaurosis 11, *ARID1B* with Coffin-Siris syndrome, *PRDM8* with progressive myoclonic epilepsy-10, *PCNT* with microcephalic osteodysplastic primordial dwarfism type II, *CITED2* with ventricular septal defect 2, and *VAX1* with microphthalmia, cleft lip and palate, and agenesis of the corpus callosum. Among potential imprinted genes, of key importance could be those that impact gene regulation. Examples include genes for transcription factors and genes that affect chromatin structure. Notably, there are many examples of syndromes that arise from mutations in transcription factor genes [13]. In that context, potential imprinted genes, discovered by my approach, include *CITED2*, *ZBTB2*, and *VAX1*.

## Results

To create density-plots, I used a Perl script that counted and reported the number of ZFBS-morph overlaps along a sliding window consisting of 850 bases. The script ignored isolated occurrences in the window and hence removed background noise. I selected the window size by trial and error as described in [14]. Large windows tended to produce false peaks. Small windows tended to give peaks with spikey appearance. In preliminary assessments, I found that the density peaks covering 2 ZFBS-Morph overlaps could be false or true-positive. Therefore, to locate candidate ICRs, I primarily examined the ‘robust’ peaks encompassing 3 or more ZFBS-Morph overlaps. In evaluations, I asked whether I could locate the known ICRs/gDMRs within relatively long genomic DNA sections. Concurrently, I inspected the DNA for peaks that may reflect the genomic positions of candidate ICRs. By displaying density-plots at the UCSC browser, I could obtain enlarged views to investigate the positions of the candidate ICRs with respect to genes, transcripts, and the CpG islands (CGIs). Additional assessments included determining whether the predicted imprinted genes corresponded to candidate imprinted genes discovered by studying allele-specific expression in tissues obtained from human term placenta [15]. I also inspected several of the density peaks in the context of their positions with respect to short clinical variants producing disease-states or developmental anomalies.

### Density-plots predicted ICRs for parent-of-origin specific expression of several experimentally identified candidate imprinted genes

To investigate the robustness of my approach, initially I assessed the positions of density peaks with respect to candidate imprinted genes listed in a previous report [15]. That list was produced from high-throughput examinations of allele-specific expression in tissues from human term placenta [15]. In my studies, I examined whether any of the listed genes corresponded to potential imprinted genes predicted by my approach. Among the candidates, the UCSC genome browser did not find *MGC16597* and *MGC24665*. Among the remaining candidates, density-plots revealed peaks within or in the vicinity of *PRDM8*, *SQSTM1*, *NM_006031*, *TJP2*, *CDK2AP1*, *MYH7B*, and *MAN2C1* (Table 1).

**Table 1:** Predicted ICRs for candidate imprinted gene identified by experimental techniques [15]

chr4:81,122,001-81,124,000	*PRDM8*
chr5:179,233,001-179,234,500	*SQSTM1*
chr7:158,713,001-158,714,500 ^#^	*WDR60*
chr7:158,621,001-158,622,500 ^&^	
chr9:71,736,276-71,737,400 ^#^	*TJP2*
chr12:123,755,871-123,756,470 ^#^	*CDK2AP1*
chr15:75,660,396-75,661,095 ^#^	*MAN2C1*
chr20:33,575,636-33,576,535 ^#^	*MYH7B*
chr21:47,823,001-47,824,200 chr21:47,832,551-47,833,700	PCNT^*^

^#^ This density peak encompasses 2 ZFBS-morph overlaps. Therefore, it could be a true or a false positive

^&^ This position could be a better candidate ICR For *WDR60*

^*^ This locus includes two robust density peaks

**Fig. 1:**
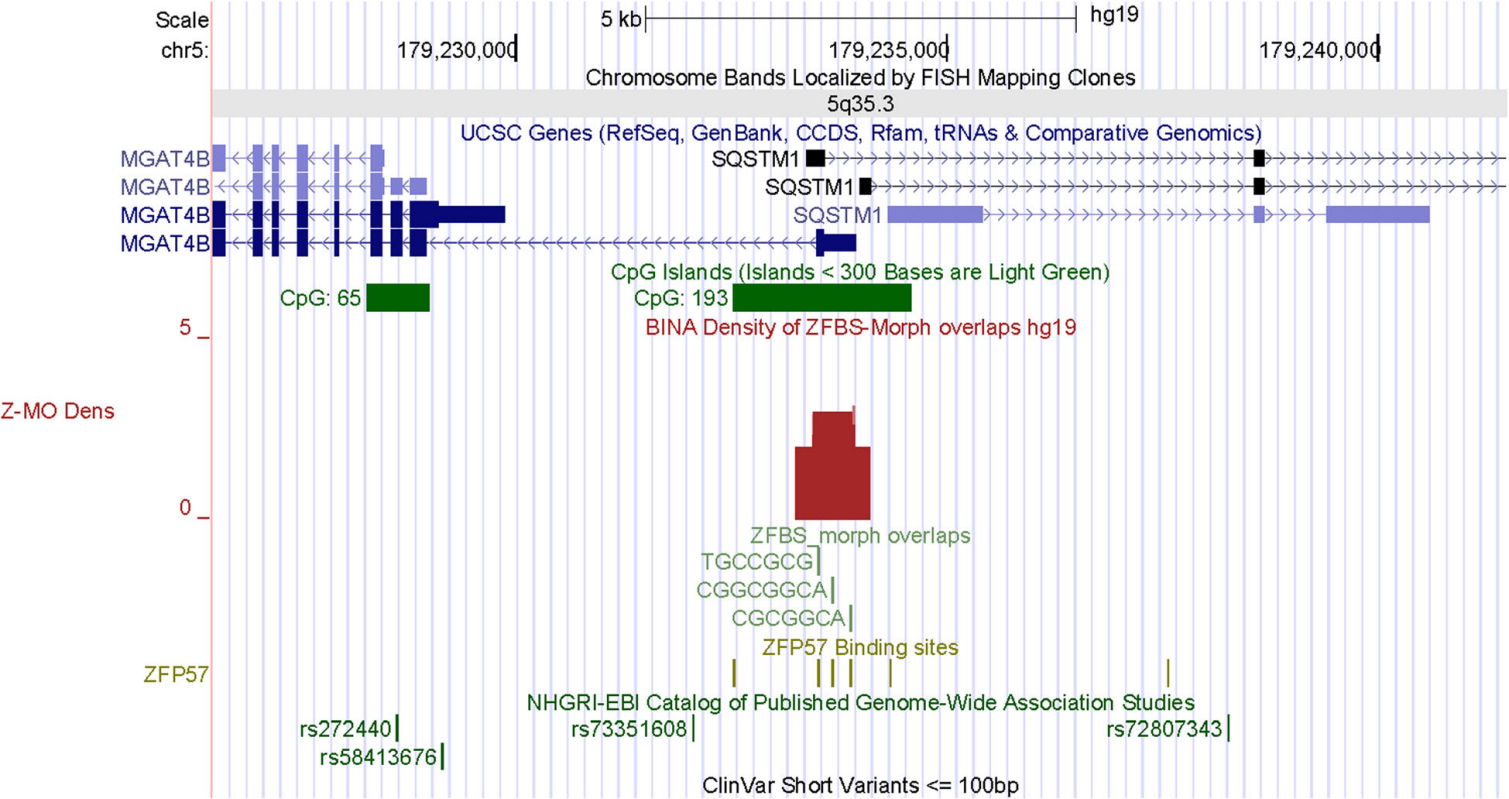
A candidate ICR mapping to overlapping transcripts (*MGAT4B* and *SQSTM1*). High-throughput experimental analysis has identified *SQSTM1* as a candidate imprinted gene [15]. The density-plots include a peak corresponding to a candidate ICR for imprinted expression of *SQSTM1*. Furthermore, the position of this ICR predicts parent-of-origin specific expression for the longest transcript produced from *MGAT4B*

In the *PRDM8* locus, I observed a robust intragenic density peak. This peak maps to a CGI in the last exon of several *PRDM8* transcripts (Fig. S1). This finding agrees with the correspondence of *PRDM8* to an actual imprinted gene. Next, I checked the positions of peaks in a DNA segment that included *SQSTM1*. This segment contains two overlapping genes (*MGAT4B* and *SQSTM1*). A relatively long CpG island encompassed the TSSs of three *SQSTM1* transcripts and the longest *MGAT4B* transcript (Fig. 1, Table 1, and Table 2). In density-plots, I observed an intragenic density peak within that island. Thus, my strategy revealed a candidate ICR regulating allele-specific expression of *SQSTM1*. Furthermore, while lending support for the correspondence of *SQSTM1* to a genuine imprinted gene, density-plots also located a potential imprinted transcript produced from *MGAT4B* (Fig. 1, Table 2). For the gene listed as *NM_006031* in reference [15], the UCSC genome browser displayed the actual gene (*PCNT*). Within that locus, density-plots revealed two very robust peaks supporting that *PCNT* could be a genuine imprinted gene (Fig. S2). For three loci, I observed peaks covering 2 ZFBS-Morph overlaps. Therefore the corresponding candidate ICRs could be true or false positive [16].

**Table 2:** Genomic positions of candidate ICRs and imprinted genes deduced in this report

chr5:179,233,001-179,234,500	*MGAT4B*
chr6:151,711,301-151,713,300	*ZBTB2*
chr6:143,832,201-143,833,400	*FUCA2*
chr6:139,693,901-139,695,200	*CITED2*
chr6:157,098,501-157,101,000	ARID1B^*^ and *LOC115308161*
chr6:150,584,501-150,586,000	*PPP1R14C* and possibly *IYD*
chr6:147,829,201-147,830,300	*SAMD5*
chr7:158,621,001-158,622,500	*ESYT2*
chr7:130,418,401-130,419,500	*KLF14*^$^
chr7:128,045,491-128,046,440	*IMPDH1*
chr10:118,896,501-118,897,800	*VAX1*

^*^ A known imprinted gene in mouse [29]

^$^ a known human imprinted gene [30]

For *FLJ10300*, the genome browser showed a sequence (*AK001162*) mapping to *WDR60*. According to OMIM, this gene is associated with Short-rib thoracic dysplasia 8 (SRTD8) with or without polydactyly [17]. Within *WDR60*, I observed a density peak covering 2 ZFBS-Morph overlaps (Fig. S3). However, in plots I noticed a very robust density peak mapping to the 5′ end of *ESYT2* (Fig. S3). This peak also could be a candidate ICR for regulating parent-of-origin expression from *WDR60*. Similarly, for several of the listed candidate imprinted genes [15], I noticed density peaks far upstream or downstream of their TSSs. Examples include *XRRA1*, *CD151*, and *VPS11*. Thus, overall, density peaks predicted ICRs for several candidate imprinted genes discovered by experimental and computational strategies. Furthermore, my results revealed that several of these genes corresponded to potential imprinted genes predicted by my approach.

### In density-plots, robust peaks pinpointed several of the known gDMRs/ICRs and revealed candidate ICRs and imprinted genes

**Fig. 2:**
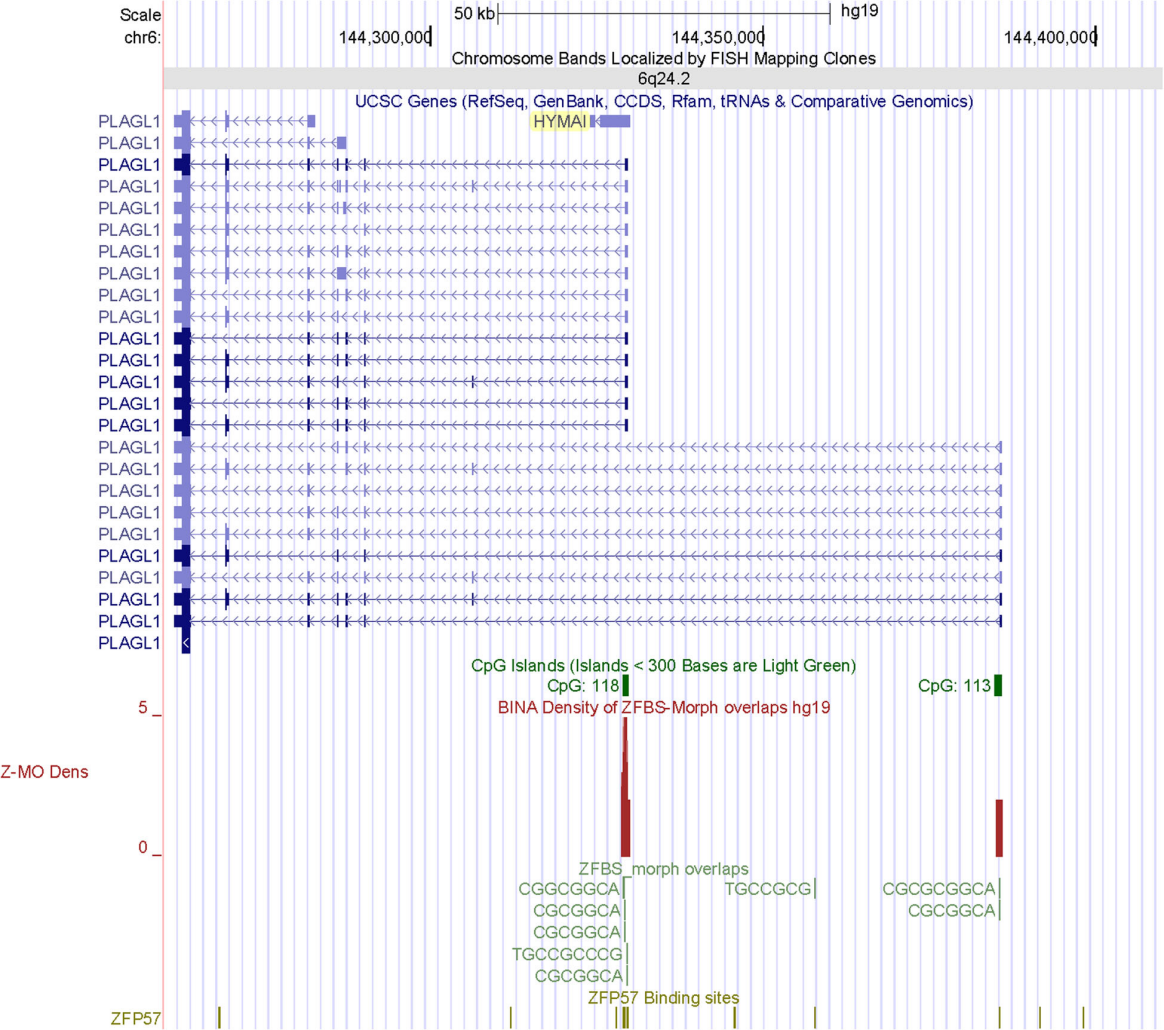
The position of a robust density peak locating the ICR in the *PLAGL1* locus. In descending order, tracks display the positions of the chromosomal bands (gray), genes and transcripts (blue), CGIs (green), peaks in the density-plots (maroon). The sequences of ZFBS-Morph overlaps are shown in pack format (hunter green), the canonical ZFP57 in dense format (olive green)

To further assess the validity of my strategy, initially I inspected the density-plot obtained for Chr6. This chromosome includes the known intragenic ICR in the *PLAGL1* locus [18, 19]. This ICR is ~ 1 kb and maps to a CpG Island (CpG118) that encompasses the TSSs of a noncoding RNA gene (*HYMAI*) and the *PLAGL1* transcript (*ZAC1*) expressed from one of two parental alleles [18, 20]. In closeup views of the locus, clearly apparent is a single robust intragenic density peak in CpG118. Thus, this peak correctly located the ICR that regulates parent-of-origin specific transcription of *ZAC1* and *HYMAI* (Fig. 2).

**Fig. 3:**
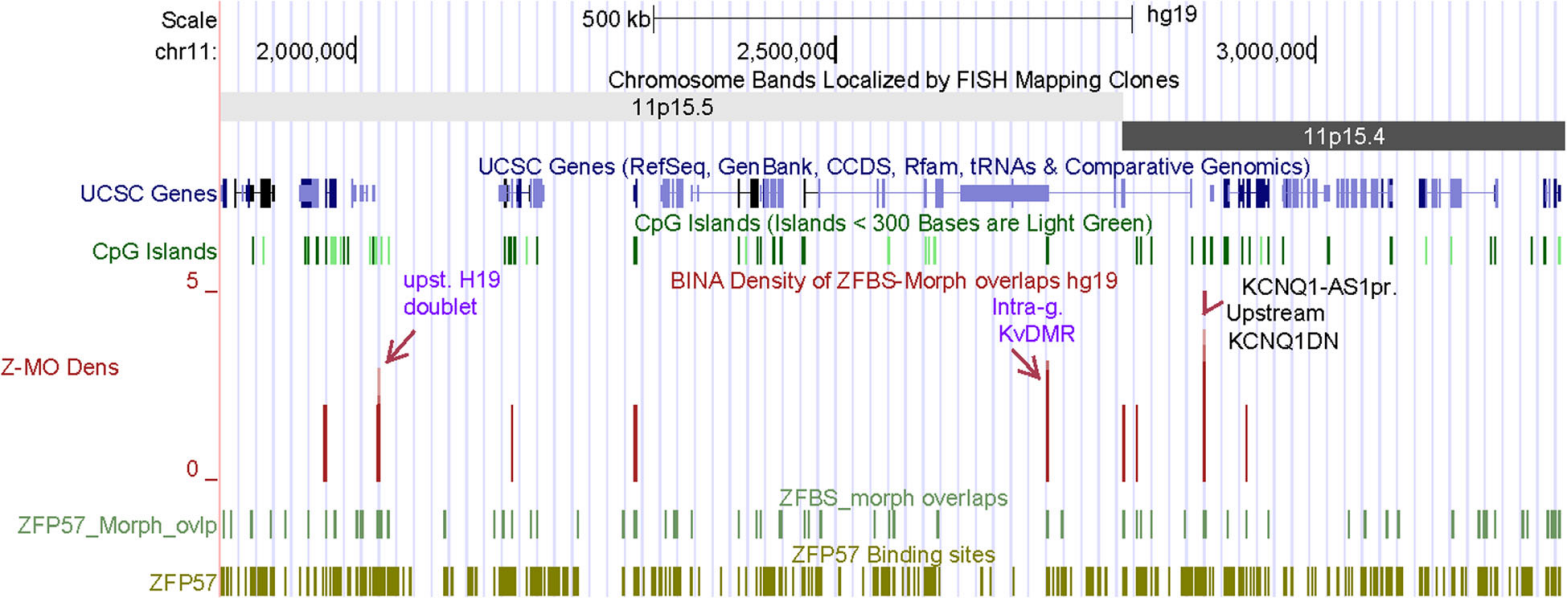
The positions of peaks in a density-plot covering 1.4 Mb long DNA. The density-plot is shown in full format. The other tracks are shown in dense-format. Purple letters denote the known ICRs. Black letters denote an intergenic candidate ICR

Next, I inspected the density-plot obtained for Chr11 to assess the positions of peaks with respect to two of the well-known imprinted domains. In Chr11, the telomeric imprinted domain 1 includes a noncoding RNA gene (*H19*) and genes that encode insulin-like growth factor 2 (*IGF2*) and insulin (*INS*). The imprinted domain 2 is relatively long and encompasses three noncoding RNA genes (*KCNQ1OT1*, *KCNQ1-AS1* and, *KCNQ1DN*) and ~ 10 protein coding genes including *KCNQ1*, *CDKN1C*, *SLC22A18*, *PHLDA2*, and *OSBPL5* [21]. In initial evaluations of plots, I inspected several long DNA sections encompassing both the *H19* – *IGF2* and the *KCNQ1* imprinted domains. In a 1.4 Mb DNA, I observed 3 robust density peaks and several peaks covering 2 ZFBS-morph overlaps (Fig. 3). One of the robust peaks appeared as a doublet and mapped to the imprinted domain 1. The other two robust peaks are in imprinted domain 2 (Fig. 3).

In domain 1, a single gDMR/ICR regulates transcription of *H19* selectively from the maternal allele and expression of *IGF2* and *INS* from the paternal allele [22]. This ICR is upstream of *H19* TSS and often is described in the context of several unique repeats [23] and sites predicted to bind the transcription factor CTCF [24]. Previously, I performed detailed analyses to determine the accuracy of the mapped repeats and the predicted CTCF sites [25, 26]. Mostly, results of my analyses agreed with the reported repeat positions [23]. However, results of the ENCODE data did not support the existence of the predicted CTCF site 5. Additionally, based on the ENCODE data, I discovered a new CTCF site that mapped to a chromatin boundary consisting of CTCF, RAD21, and SMC3 –for details see reference [25]. The new CTCF site (numbered 8) is in a CGI upstream of *H19* TSS. Furthermore, in closeup view of density-plots, I noticed two closely-spaced density peaks locating correctly the ICR in the imprinted domain 1 (Fig. S4).

Domain 2 is relatively long and includes the ICR (KvDMR1) that regulates expression of several imprinted genes [21, 27]. This ICR is intragenic and is located in the vicinity of *KCNQ1OT1* TSS. In closeup views, I observed a robust density peak within the CpG island that encompasses the *KCNQ1OT1* TSS of and the KvDMR1 (Fig. S5). Another robust density peak is between two noncoding RNA genes corresponding to *KCNQ1DN* and *KCNQ1-AS1* (Fig. 3). *KCNQ1DN* is an imprinted transcript within the WT2 critical region [28]. *KCNQ1-AS1* was reported in the genome assembly NR_130721.1. Even though the function of *KCNQ1-AS1* is unknown, it is worth noting that it is transcribed antisense with respect to *KCNQ1OT1*. Therefore, the expression of *KCNQ1-AS1* might be a mechanism to impede leaky production of *KCNQ1OT1* in a subset of human tissues.

### Density-plots offer a strategy to discover potentially novel imprinted genes, candidate ICRs, and to determine their possible associations with clinical abnormalities

**Fig. 4:**
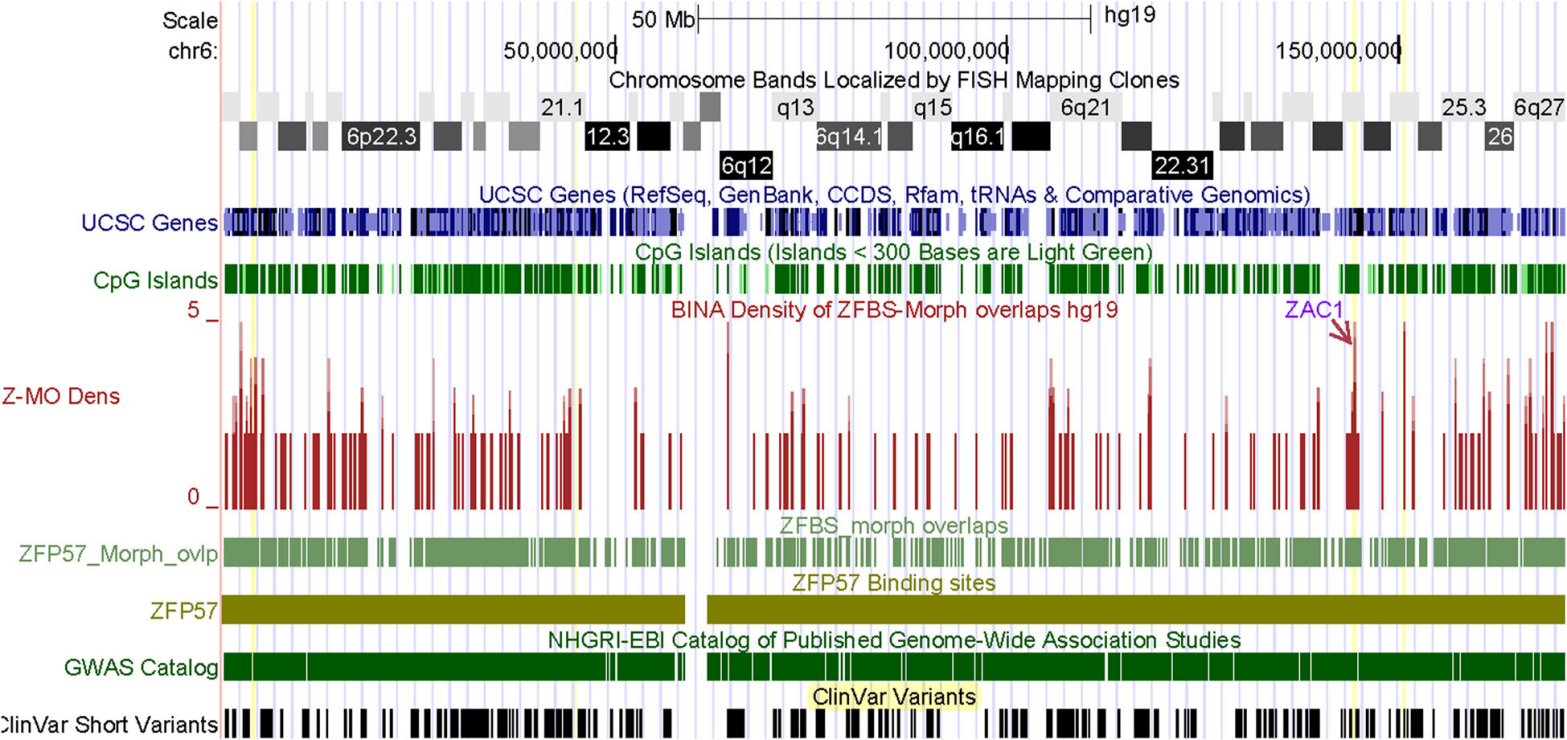
The positions of density peaks along the entire Chr6. At the UCSC genome browser, one could examine the peak positions with respect to genes, in the context of clinical variants, and Genome-wide Association Studies (GWAS). These tracks facilitate examining the potential imprinted genes in the context of diseases, genetic abnormalities, and genome-wide variants associated with a trait. Note that within nearly 172 Mb DNA, clearly discernable is a very robust peak corresponding to the ICR of *ZAC1*. Along the chromosome are additional robust peaks for candidate ICRs

In humans, defects in imprinted genes could cause severe diseases and developmental anomalies [1]. Therefore, I explored whether density-plots could help with finding unknown imprinting genes and whether any of these genes could be associated with genetic disorders. In exploratory studies, I also inspected peak resolutions in plots obtained for entire chromosomal DNA sequences. For example, examine the plot displaying the position of peaks in Chr6 (Fig. 4). The density peaks and short clinical variants are primarily in gene rich regions. I noticed that Chr6 includes several robust peaks. Notably, one of the discernable peaks corresponds to the ICR of *ZAC1* and *HYMAI* in the *PLAGL1* locus (Fig. 4).

### A relatively long DNA sections from Chr6q includes candidate ICRs for several potential imprinted genes

**Fig. 5:**
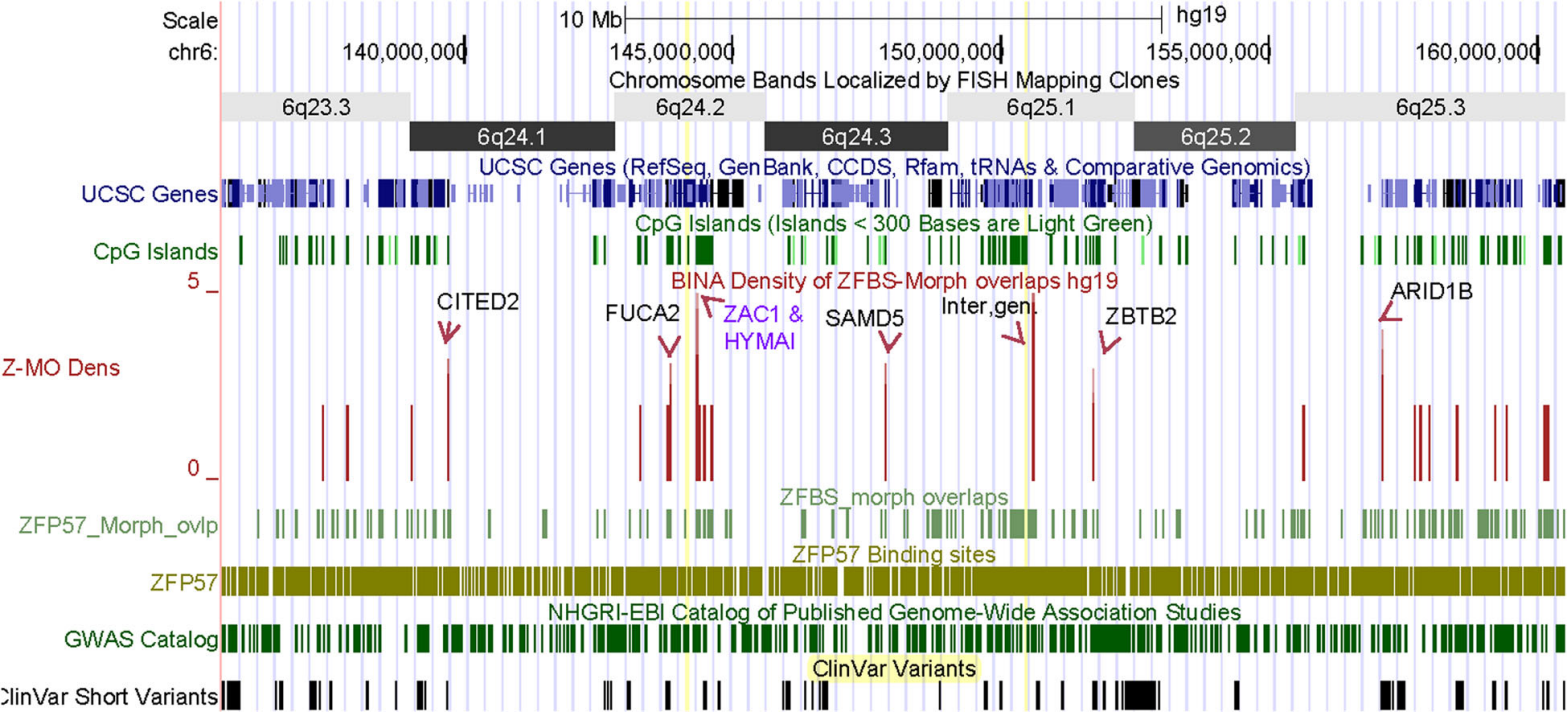
Discovering candidate ICRs and novel imprinting genes in Chr6. The displayed chromosomal section includes several density peaks dispersed across several bands. One of the robust peaks corresponds to the ICR of *ZAC1* and *HYMAI*. The remaining robust peaks define the positions of candidate ICRs for potential imprinted genes or transcripts

For close-inspections of plots with respect to clinical variants, I selected several relatively long DNA sections that included a known ICR and candidate ICRs for potential imprinted genes. Initially I zoomed in a segment in Chr6 that included the known ICR of *ZAC1*. The displayed segment contains 25 Mb DNA encompassing several chromosomal bands (Fig. 5). Even within such a long genomic DNA section, the density peaks appear clearly resolved. In that view, I observed 7 robust density peaks (1 per ~ 3.6 Mb) demonstrating that robust peaks occurred sporadically in human genomic DNA. One of the robust peaks pinpointed the known intragenic ICR of *ZAC1* and *HYMAI* (Figs. 4 and 5). The remaining 6 reflect the positions of candidate ICRs dispersed in various genomic locations. For example, in Chr6q25.1 I noticed an intergenic candidate ICR between *PPP1R14C* and *IYD*. Additional candidates ICRs are dispersed along the 25 Mb DNA mapping to *CITED2*, *FUCA2*, *SAMD5*, *ZBTB2*, and *ARID1B* (Fig. 5, Table 2).

Chr6q24.1 includes the candidate ICR for *CITED2*. The corresponding density peak is in an intragenic CpG island in the last *CITED2* exon (Fig. S6). CITED2 (CBP/p300-interacting transactivator 2) regulates transcription. Absence of *Cited2* in mouse embryos caused congenital heart disease by perturbing left-right patterning of the body axis [31]. In Chr6q24.2, a candidate ICR maps to an intragenic CpG island near the 5′ end of the longest of *FUCA2* transcript (Fig. S7). Genome-wide analyses have identified *FUCA2* and *IL-18* as novel genes associated with diastolic function in African Americans with sickle cell disease [32]. A previous study deduced that *FUCA2* was biallelically expressed [19]. As listed in a zip file, the analyzed SNP (rs72992630) and primers were from an intragenic *FUCA2* exon [19]. In contrast, the candidate ICR is in a CpG island (CpG52) that encompasses the 1st exon of the longest of *FUCA2* transcript. This exon is at ~ 9.5 kb upstream of the analyzed SNP and the primers selected for expression analyses; for details see (Fig. S7).

Density-plots include a candidate ICR in chr6q24.3. The corresponding peak maps to a CpG island that encompasses *SAMD5* TSS (Fig. S8). A study found that *SAMD5* was overexpressed in prostate cancer and had powerful prognostic ability for predicting post-operative biochemical recurrence after radical prostatectomy [33]. In Chr6q25.1, a candidate ICR maps to a CpG island at the 5′ end of *ZBTB2* (Fig. S9). ZBTB2 binds DNA and is among the master regulators of the p53 pathway [34]. In mouse embryonic stem cells, ZBTB2 dynamically interacted with nonmethylated CpG island promoters and regulated differentiation [35]. In colorectal cancer, the abnormal forms of *ZBTB2* increased cell proliferation [36].

**Fig. 6:**
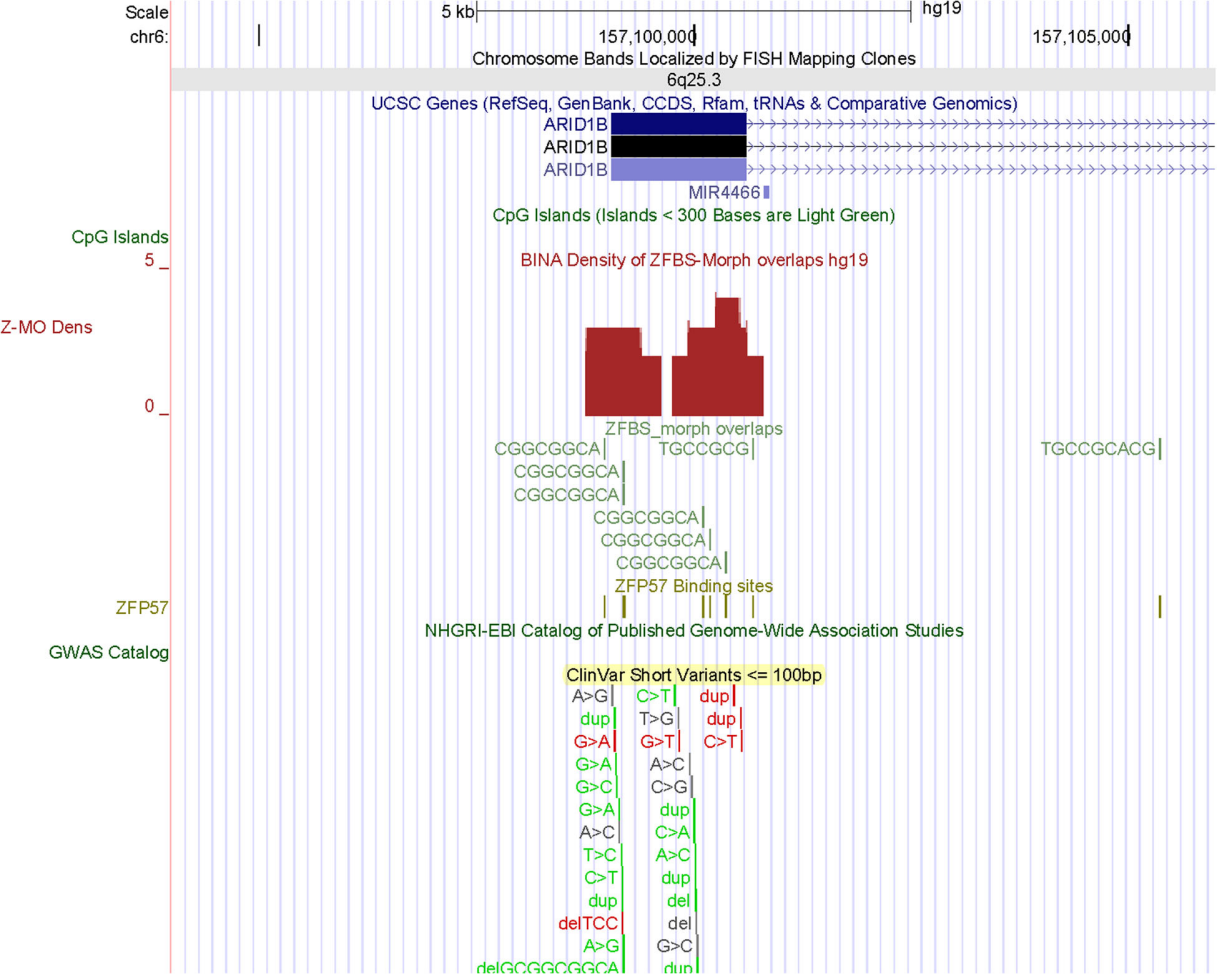
A candidate ICR regulating parent-of-origin specific expression of *ARID1B*. The official name of *ARID1B* is *KDM5B*. In mouse *Arid1b* is a known imprinted gene [29]

**Fig. 7:**
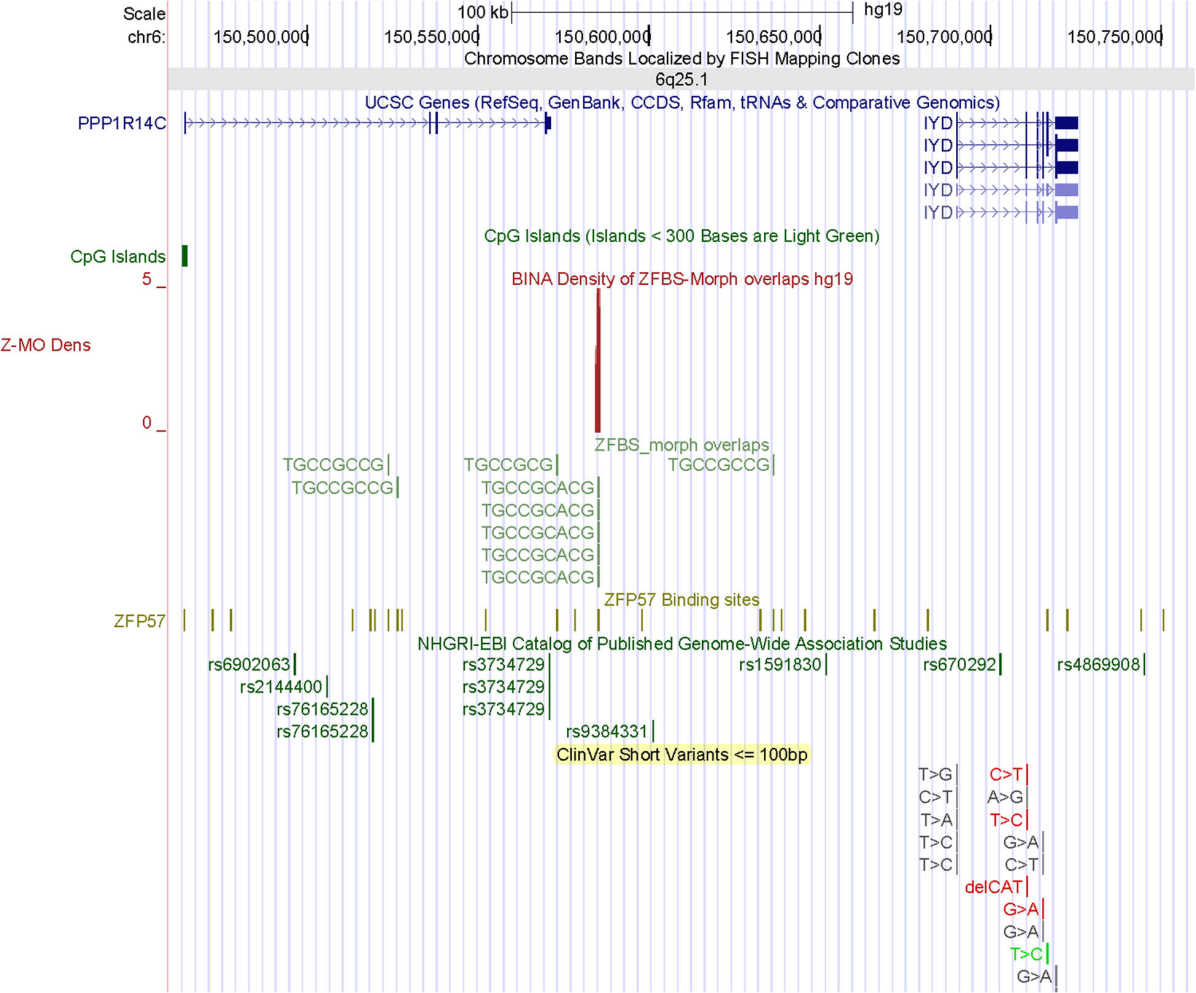
An intergenic candidate ICR regulating parent-of-origin specific expression of *PPP1R14C* and *IYD*. GWAS identified several potentially significant SNPs in *PPP1R14C*. Thyroid dyshormonogenesis-4 (TDH4) is caused by homozygous mutations in *IYD*. Patients with this defect lack the ability to deiodinate radiolabeled monoiodotyrosine and diiodotyrosine [40]

Chr6q25.3 includes 1 candidate ICR (Fig. 5). This ICR corresponds to 2 density peaks (Fig. 6). One peak encompasses *ARID1B* TSS. The other maps to the *ARID1B* 1st exon. Since *Arid1b* is a known imprinted gene in mouse [29], from my data one could deduce that its human ortholog also is an imprinted gene. *ARID1B* encodes an enzyme that removes H3K4 methyl-marks from chromatin [37]. In mouse, Arid1b haploinsufficiency impacted and disrupted cortical interneuron development [38]. In Chr6q25.1, a candidate ICR is between *PPP1R14C* and *IYD* (Fig. 7). The corresponding density peak is in the vicinity of the 3′ end *PPP1R14C* in a DNA segment far upstream of *IYD* TSS. PPP1R14C regulates the enzymatic activity of protein phosphatase 1. *IYD* encodes an enzyme that functions in iodide salvage in the thyroid [39]. Thyroid dyshormonogenesis-4 (TDH4) is caused by homozygous mutations in *IYD*. Patients with this defect lack the ability to deiodinate radiolabeled monoiodotyrosine and diiodotyrosine [40]. Notably, the thyroid hormone pathway includes an enzyme (DIO3) encoded by an imprinted gene [41].

### A DNA section from Chr7q includes a candidate ICR for a known imprinted gene (*KLF14*) and a candidate ICR for a potential imprinted gene (*IMPDH1*)

**Fig. 8:**
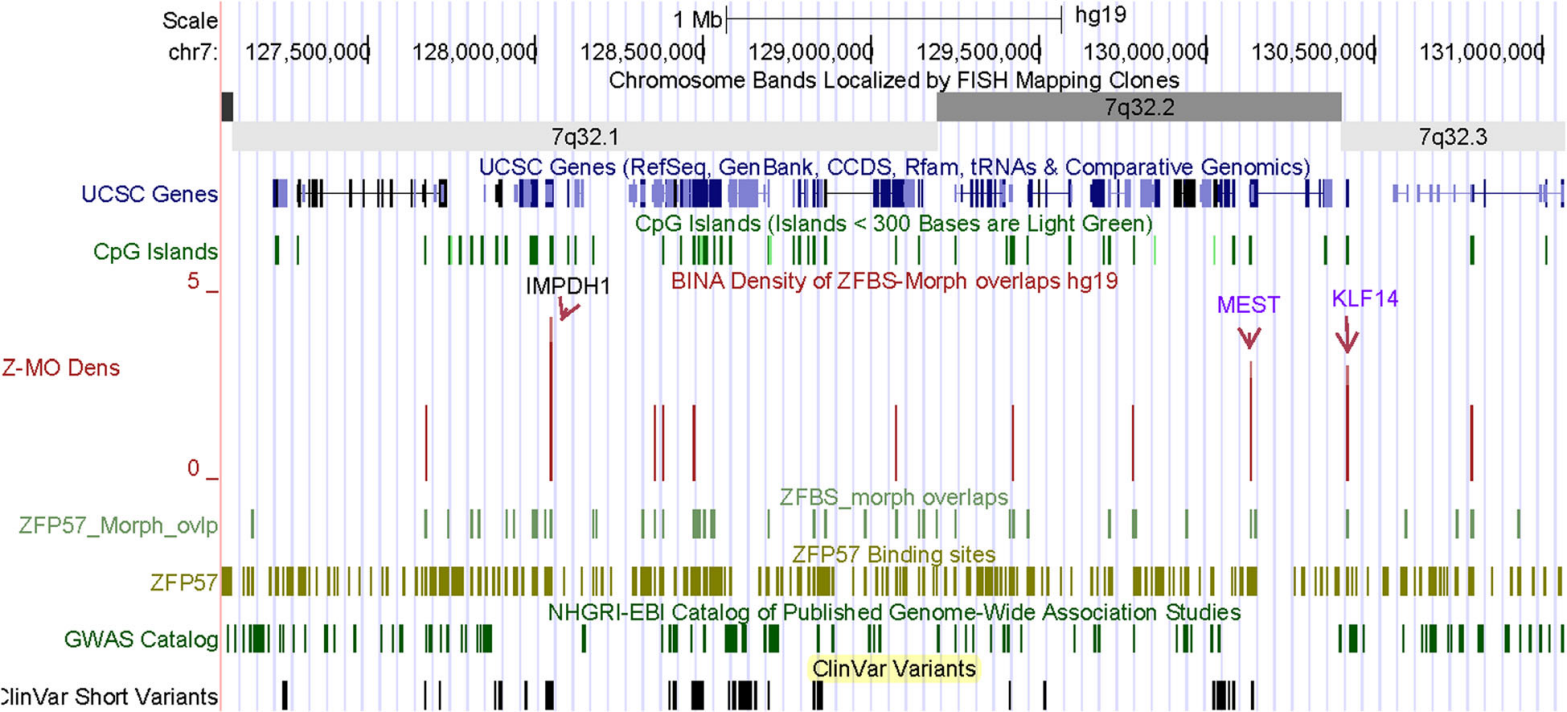
A long DNA section from Chr7q. The displayed section contains the ICR in the *MEST* locus, a known imprinting gene (*KF14*), and a candidate ICR for a potential imprinted gene (*IMPDH1*)

Chr7 contains several known imprinted genes [42, 43]. From Chr7qA, I selected a 4 Mb long DNA covering 3 robust density peaks (1 per 1.3 Mb). These peaks map to *IMPDH1*, *MEST*, and *KLF14* (Fig. 8). The *MEST* and *KLF14* loci includes known imprinted transcripts [30, 44]. In the *MEST* locus, an intragenic ICR regulates parent-of-origin-specific expression of a subset of *MEST* transcripts and *MESTIT1* –a noncoding RNA gene [45, 46]. An enlarged view of density-plots shows a peak within an intragenic CpG island that encompasses the TSSs of both *MEST* and *MESTIT1* (Fig. S10). Thus, a peak at that position correctly pinpointed the ICR in the *MEST* locus. The displayed view also shows the peak that corresponds to *KLF14* (Fig. S10). *KLF14* is a known human imprinted gene. It is the first example of an imprinted gene that has undergone accelerated evolution in the human lineage [30]. To date, I have not found a report locating the ICR of *KLF14*. Notably, density plots predicted a candidate ICR within a CpG island that encompasses *KLF14* TSS (Fig. S10, Table 2).

**Fig. 9:**
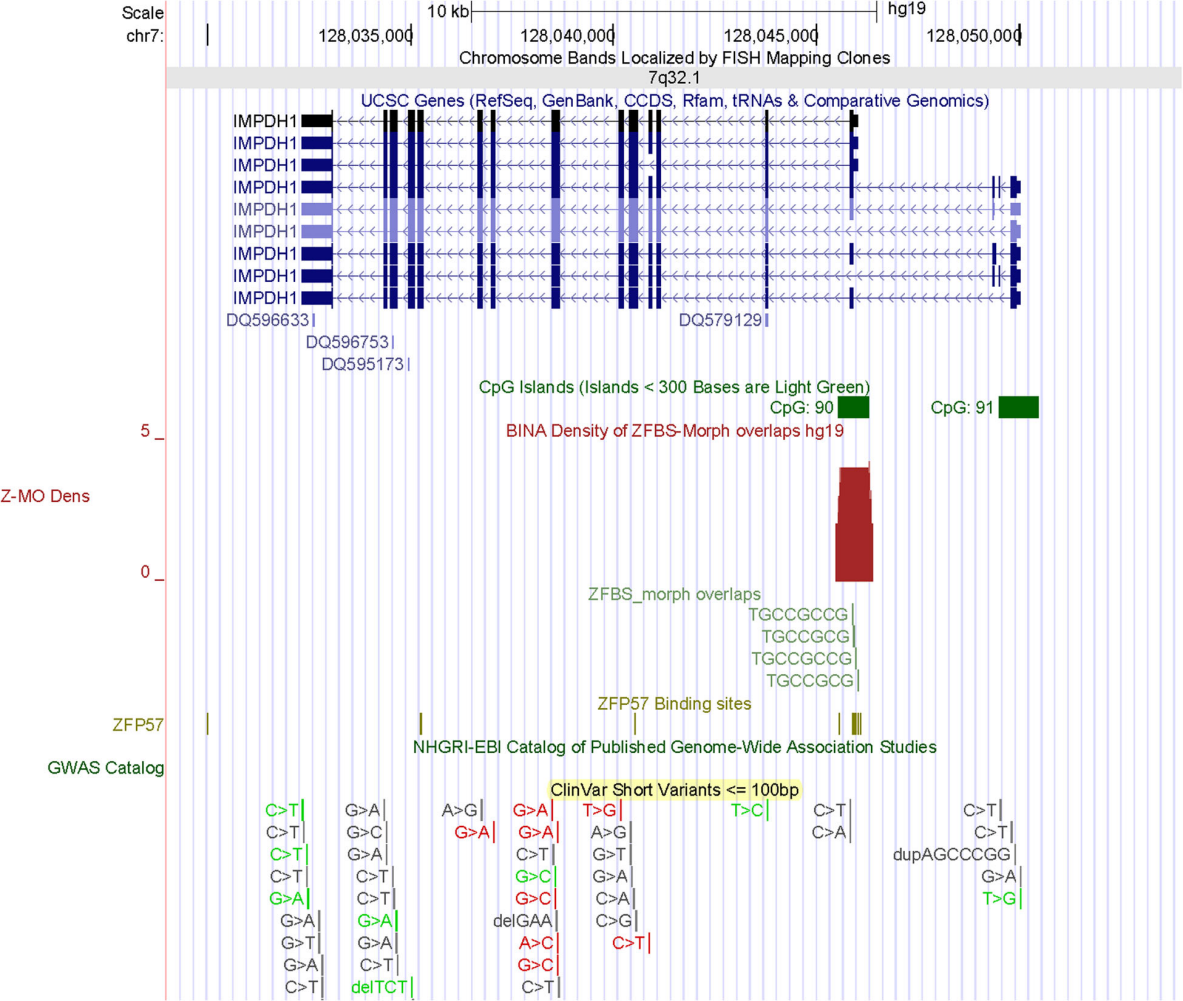
A candidate ICR in the *IMPDH1* locus. The corresponding peak maps to a CGI that encompasses TSSs of several intragenic *IMPDH1* transcripts

Next, I obtained a closeup view to inspect the position of a candidate ICR that mapped to *IMPDH1* (Fig. 9). As observed for the *MEST* locus, this ICR is intragenic and maps to a CpG island that encompasses the TSSs of several short *IMPDH1* transcripts (Fig. 9). Even though *IMPDH1* is expressed in many tissues, its predominant transcripts are produced in the inner segment and synaptic terminals of retinal photoreceptors [47]. The IMPDH proteins form active homo-tetramers that catalyze the rate-limiting step for de novo guanine synthesis by converting inosine monophosphate to xanthosine monophosphate [47]. Deleterious mutations in *IMPDH1* cause Leber congenital amaurosis 11. Manifestations of this genetic anomaly include a group of early-onset childhood retinal dystrophies [48].

### A long DNA section from Chr10q includes a candidate ICR for a potential imprinted gene (*VAX1*)

**Fig. 10:**
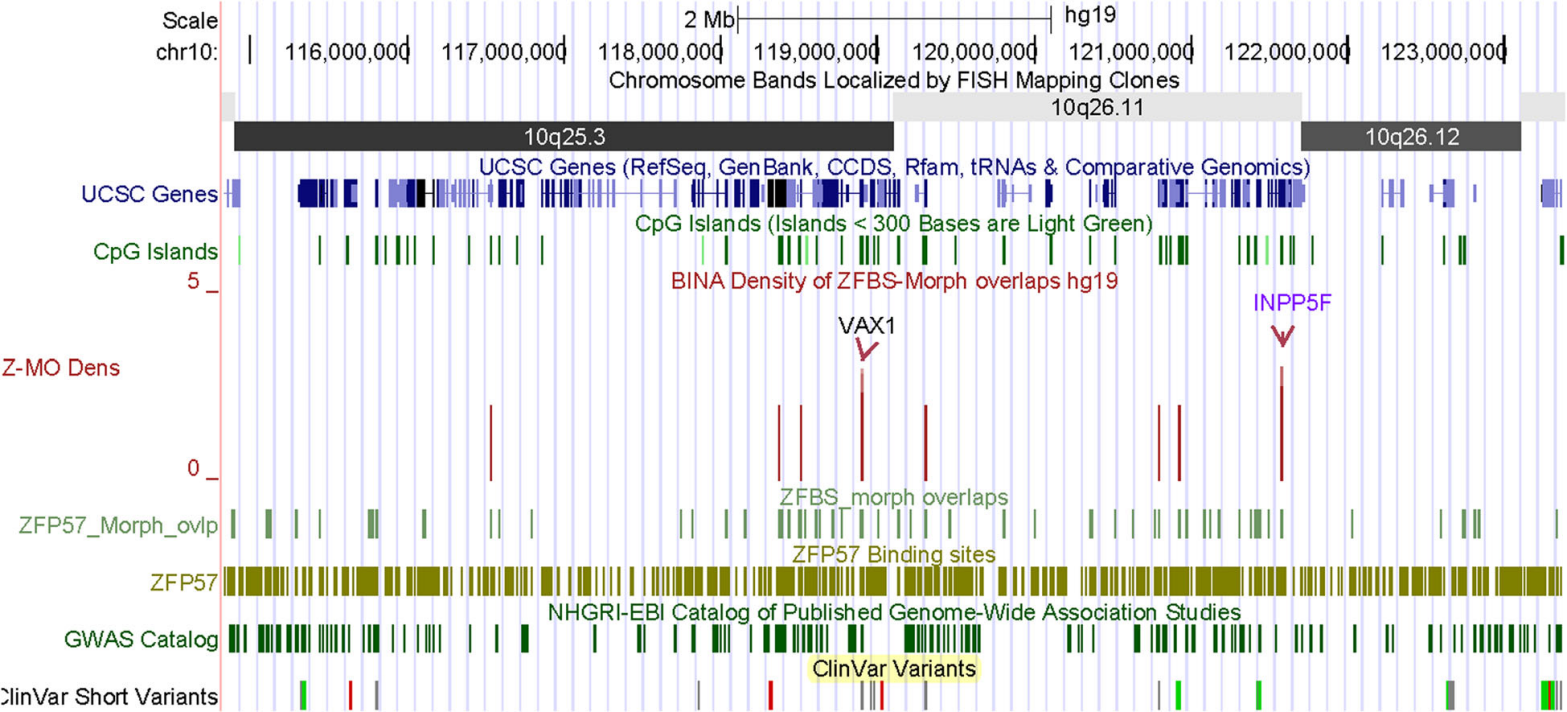
A long DNA section encompassing two robust density peaks. One of the peaks maps to the known ICR in INPP5F locus

**Fig. 11:**
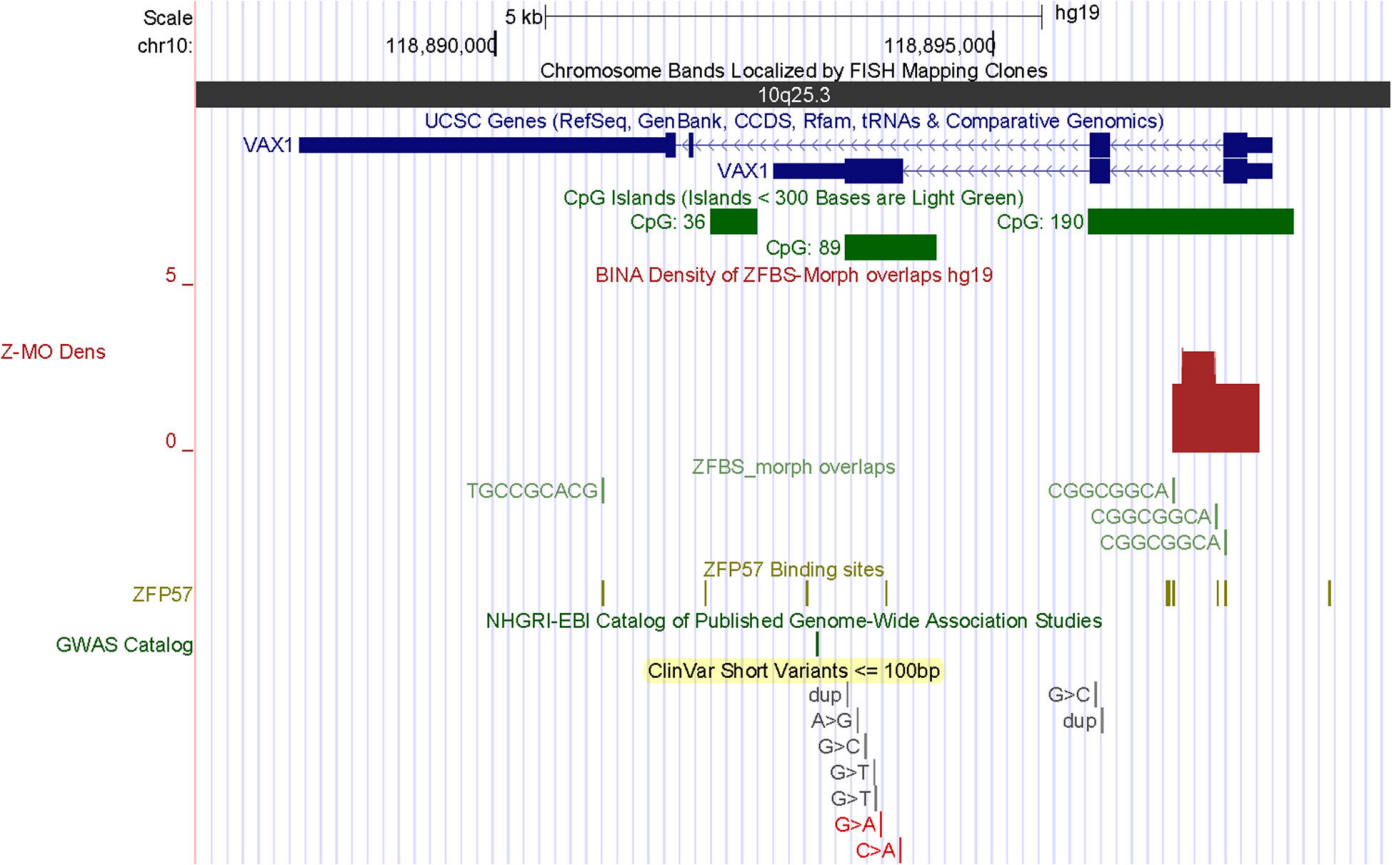
A candidate ICR regulating expression of a potential imprinted gene. The corresponding density peak is in a CGI that encompasses TSSs of *VAX1* transcripts

In Chr10q, a nearly 8.6 Mb DNA encompasses several chromosomal bands and 2 robust density peaks (1 per 4.3 Mb). One of the peaks is within the *INPP5F* locus. The other maps to *VAX1* (Fig. 10). From *INPP5F* are produced several transcriptional variants. In mouse, one of the variants (*Inpp5f_v2*) is imprinted in the brain [49]. The TSS of *Inpp5f_v2* is within a differentially methylated CpG island [49]. In closeup views of density-plots, I observed an intragenic density peak that pinpointed the ICR for parent-of-origin specific expression of human *INPP5F_v2* (Fig. S11). A candidate ICR is in a CpG island that encompasses TSSs of 2 *VAX1* transcripts (Fig. 11). This gene encodes a transcription factor with a homeobox for binding DNA. *VAX1* is expressed in the pituitary, hypothalamus, and testis [50]. A study implicated two homozygous mutations in *VAX1* causing microphthalmia associated with cleft lip and palate and agenesis of the corpus callosum [51].

### Examination of candidate ICRs with respect to several previously predicted imprinted genes

The literature includes various computational strategies for prediction of novel imprinted genes. Examples include sequence or epigenetic features [52, 53]. I looked at a listing of predicted human imprinted genes [54] to assess whether density-plots included candidate ICRs in their vicinity. I located candidate ICRs for several of the predicted genes (Table 3). Several of these ICRs corresponded to density peaks encompass 2 ZFBS-morph overlaps. Therefore, I am not confident whether the predicted ICRs could be true or a false-positive (Table 3). I did not find a candidate ICR for several of the predicted imprinted genes including *HOXA2*, *HOXA3*, *HOXA5*, *HOXC9*, *HOXC4*, *IFITM1*, *PKP3*, *SLC26A10*, *CDH15*, *RASGRF1*, and *ZRSR1*.

**Table 3:** Examination of several predicted human imprinted genes with respect to candidate ICRs observed in density-plots

ICRs Positions in the build hg19	Neighboring transcripts
chr7:42,275,801-42,277,360	*GLI3*
chr7:150,783,541-150,784,530 ^*^	*FASTK*
chr7:27,169,571-27,170,500 ^#^	*HOXA4*
chr7:27,224,001-27,224,600	*HOXA11*
chr7:27,291,381-27,292,450 ^&^	*EVX1*
chr6:105,387,976-105,388,975 ^+^	*LIN28B*
chr7:77,648,446-77,649,555 ^#^	*MAGi2*

^*^ This predicted ICR could be a true or a false positive. It is ~ 5400 bp upstream of *FASTK* at the 5′ end of *AGAP3*, transcript variant 3

^#^ This predicted ICR encompasses 2 ZFBS-morph overlaps. Therefore, it could be a true or a false positive

^&^ This density predicted ICR is far downstream *EVX1* and upstream of *EVX1-AS*

^+^ This predicted ICR is far upstream of *LIN28B* and near *LIN28B-AS1*

## Discussion

The discovery of unknown ICRs would facilitate pinpointing the genes in their vicinity and thus uncovering novel imprinted genes. Furthermore, examination of the imprinted genes in the context of clinical variants gives clues into their impacts on human embryonic development, disease states, and genetic anomalies including syndromes [1]. However, to date I could not find any genome-wide studies to methodically discern the genomic positions of the ICRs and to obtain nearly complete listings of novel imprinted genes and transcripts. Towards these and related goals, I developed a predictive genome-wide strategy. My approach pinpointed several of the known ICRs within relatively long DNA sections. Examples include the ICRs of *H19* – *IGF2* and *KCNQ1* imprinted domains in 1.4 Mb long DNA (Fig. 3); the ICR of *MEST* and *MESTIT1* transcripts in 4.0 Mb DNA (Fig. 8), and the ICR of I*NPP5F_V2* within a nearly 8.6 Mb DNA (Fig. 10). Even along the entire Chr6, I could discern the ICR of *ZAC1* and *HYMAI* in the *PLAGL1* locus (Fig. 4).

My strategy involves creating density-plots to detect the genomic DNA segments that contain clusters of 2 or more ZFBS-morph overlaps [14, 55, 56]. Previously, I showed that occurrences of such clusters pinpointed ~ 90% of the fully characterized ICRs/gDMRs in the mouse genome [7, 8, 57]. Even though my approach is predictive, it is based on reports demonstrating the importance of ZFP57 in maintaining allele-specific gene repression [5, 12, 58, 59]. Furthermore, my discovery of ZFBS-morph overlaps has offered mechanistic clues into why ZFP57 is selectively recruited to the ICRs but not elsewhere in the genomic DNA. Briefly, essential to genomic imprinting is a protein complex consisting of DNMT3A and DNMT3L [2, 3, 60]. This complex methylates DNA processively [4]. Since clusters of ZFBS-morph overlaps are CpG-rich, they could provide sites for DNMT3A-DNMT3L to processively methylate the ICRs. Subsequently, the methylated ZFBS-morph overlaps would recruit ZFP57 to associate selectively with ICRs to maintain parent-of-origin specific gene expression [7].

### The majority of candidate ICRs maps to CpG islands; a subset maps to specific gene transcripts

The UCSC genome browser is highly suitable for examining results of predictive methods in the context of landmarks including the positions of chromosomal bands, genes, transcripts, and the CpG islands [61]. ICR associated islands could be intergenic, encompass promoters, TSSs, and the 1st exon of genes [3, 62]. The ICRs also occur in intragenic CpG [45, 63, 64]. Similarly, in density-plots the analyzed robust peaks primarily map to CpG islands at various genomic locations. Examples include the candidate ICRs for *PPP1R14C*, *IYD*, *CITED2*, *IMPDH1*, and *VAX1* (Fig. 7, S6, 8, 9, and 11). Several of the known ICRs correspond to single transcripts or to transcriptional variants [62]. I observed similar patterns for several of the candidate ICRs. For example, the candidate ICRs for *SAMD5* and *ZBTB2* correspond to isolated transcripts (Figs. S8 and S9). The candidate ICR for *FUCA2* corresponds to the gene longest transcript (Fig. S7). The candidate ICR for *IMPDH1* encompasses intragenic transcripts (Fig. 9).

### Density-plots revealed candidate ICRs for potential imprinted genes associated with syndromes and disease states

With animal model systems, it is possible to determine whether knockout of a gene would produce a phenotype. In humans, one could examine adverse effects of anomalous loci in the context of clinical variants that produce discernable phenotypes. Examples include developmental disorders, neurological disorders, malformation of body parts, and syndromes. One could identify these phenotypes from literature surveys, the track displaying the clinical variants at the UCSC browser, or both. For example, examine the figure that displays the positions of short clinical variants with respect to peaks in the density-plot obtained for the entire Chr6 (Fig. 4). As these peaks, the clinical variants are primarily dispersed in gene-rich genomic DNA sections.

*ARID1B* is among the potential human imprinted genes identified by my approach (Fig. 6). In mouse *Arid1b* is a known imprinted gene [29]. My data predicts that human *ARID1B* also is an imprinted gene. This gene encodes an enzyme that removes activating H3K4 methyl marks from chromatin [65]. In human, deleterious variations in *ARID1B* are thought to contribute to Coffin-Siris syndrome. This abnormality is a multiple malformation syndrome characterized by mental retardation associated with coarse facial features, hypertrichosis, sparse scalp hair, and hypoplastic or absent fifth fingernails or toenails. Other features may include poor overall growth, craniofacial abnormalities, spinal anomalies, and congenital heart defects [66]. Mechanistically, within the protein-networks ARID1B interacts with a complex Known as NuRD (Nucleosome Remodeling and Deacetylase); for details see Fig. 1 in reference [7]. In the networks, NuRD is a central node for receiving or transmitting signals via protein-protein interactions. For example, while one of the subunits in NuRD (Mi-2α) interacts with TRIM28, its HDAC1 subunit interacts with MLL1, DNMT3A, DNMT3L, and H3K4 demethylases including ARID1B [7].

Several of the potential imprinted genes identified by my approach correspond to a subset of candidate-imprinted genes discovered by experimental techniques [15]. Examples include *SQSTM1*, *PRDM8*, and *NM_006031*/*PCNT* (Figs. 1, S1, and S2). A domain in PRDM8 methylates H3K9 and thus impacts the chromatin structure [67]. Monoallelic expression of *PRDM8* was detected in placental tissues [15]. Genetic studies have observed association of *PRDM8* with progressive myoclonic epilepsy-10. This recessive neurodegenerative disorder is characterized by onset of progressive myoclonus, ataxia, spasticity, dysarthria, and cognitive decline in the first decade of life [68]. PCNT (pericentrin) is a component of pericentriolar material that surrounds the two centrioles of a centrosome [69]. Absence of *PCNT* results in disorganized mitotic spindles and missegregation of chromosomes [70]. Heterozygous mutation in *PCNT* caused microcephalic osteodysplastic primordial dwarfism type II [71]. This disorder (MOPD2) is characterized by intrauterine growth retardation, severe proportionate short stature, and microcephaly. Adults with these inherited distinctive physical features have an average height of 100 cm and a brain size comparable to that of a 3-month-old baby. Otherwise, they have near-normal intelligence [70].

Potential imprinted genes discovered de novo by approach include *IMPDH1* (Fig. 9), *CITED2* (Fig. S6), *SAMD5* (Fig. S8), *ZBTB2* (Fig. S9), and *VAX1* (Fig. 11). IMPDH1 (inosine monophosphate dehydrogenase 1) catalyzes the synthesis of xanthine monophosphate [72]. This reaction is the rate-limiting step in the de novo synthesis of guanine nucleotides. Deleterious mutations in *IMPDH1* often cause Leber congenital amaurosis 11 (LCA11). This anomaly consists of a group of early-onset childhood retinal dystrophies characterized by vision loss, nystagmus, and severe retinal dysfunction [48]. Mutations in *IMPDH1* also may cause a disorder known as retinitis pigmentosa-10 (RP10). In most patients, RP10 is manifested by early onset and rapid progression of ocular symptoms, initially with night blindness in childhood [73]. The impairment tends to produce stable visual field constriction, although it may worsen very slowly over time. Another potential imprinted gene (*CITED2*) transactivates transcription through interactions with CBP/P300 [74]. Deleterious mutations in *CITED2* cause ventricular septal defect 2 (VSD2). As the most common form of congenital cardiovascular anomaly, VSD2 has affected nearly 50% of all infants with a congenital heart defect and accounts for ~ 15% of cardiac anomalies requiring invasive treatment within the first year of infant’s life [75]. Furthermore, congenital VSDs may arise alone or in combination with other cardiac malformations. In that context, it seems relevant that loss of *Cited2* in mouse causes congenital heart disease by perturbing left-right patterning of the body axis [31]. Therefore, it seems plausible that VSDs in infants might also stem from defects in left-right patterning of the body axis in the course of human embryonic development.

*SAMD5* is another potential imprinted gene discovered by approach. In the context of disease-related anomalies, a study found that *SAMD5* was overexpressed in prostate cancer and had powerful prognostic ability on predicting post-operative biochemical recurrence after radical prostatectomy [33]. Another potential imprinted gene (*ZBTB2*) is among the genes identified in approximately 15% of all colorectal cancers [36]. Abnormal forms of *ZBTB2* increased cell proliferation [36]. ZBTB2 is a transcription factor. Several members of the ZBTB family have emerged as critical factors in the lineage commitment, differentiation, and function of lymphoid cells as well as many other developmental events [76]. Furthermore, ZBTB2 is among the master regulators of the p53 pathway [34]. In mouse embryonic stem cells, ZBTB2 dynamically interacted with unmethylated CpG island promoters and regulated differentiation. Another potential imprinted gene (*VAX1*) encodes a transcription factor that controls developmental processes. The structure of VAX1 includes a homeodomain for binding DNA. In mouse, this homeobox-containing gene is expressed in the developing anterior ventral forebrain [77]. *Vax1* expression was observed in the pituitary, hypothalamus, and testis [50]. From studies of 70 patients, a report found associations of two homozygous mutations in *VAX1* with microphthalmia, cleft lip and palate, and agenesis of the corpus callosum [51]. For an overview [78].

## Conclusion

In this report, I have offered a predictive genome-wide strategy to discover candidate ICRs and novel imprinted genes. I gave evidence for robustness of my strategy by pinpointing several of the well-known ICRs in relatively long DNA sections. I also gave examples showing that my strategy predicted ICRs for several of the candidate imprinted genes discovered by experimental strategies. The finding that several of the potential imprinted genes impact developmental processes, lends additional support for the robustness of my approach. I also covered examples of how I could deduce the phenotypes of the potential imprinted genes discovered by my approach. Nonetheless, only experimental validations could demonstrate the strength of my approach. Therefore, I offer links for accessing and downloading my data on the positions of ZFBS and ZFBS-Morph overlaps [55], peaks in density-plots [56], and the MLL1 morphemes in the build hg19 of the human genome [79]. Links are also available for accessing data pertaining to the build mm9 of the mouse genome [16, 57, 80].

## Methods

### Marking the genomic positions of ZFP57 binding site and the ZFBS-morph overlaps

From the UCSC genome browser, I downloaded the nucleotide sequences reported for the build hg19 of the human genome. Next, I created two texts files: one file containing the nucleotide sequences of the ZFBS-Morph overlaps [8]; the other the hexameric ZFP57 binding site [5]. Using a Perl script, initially I determined the genomic positions the ZFBS-Morph overlaps along the chromosomal DNA sequences. That script opened the file containing the nucleotide sequence of a specified chromosome and the file containing the sequences of the ZFBS-Morph overlaps. After that step, the script moved along the DNA to report the genomic positions of the overlaps. Next, I wrote a subroutine to combine the outputs obtained for various chromosomes. Another subroutine produced a file to create a custom track for displaying the genomic positions of the ZFBS-Morph overlaps at the UCSC genome browser. I followed similar procedures to obtain a file to display the genomic positions of the hexameric ZFP57 binding site at the UCSC genome browser [8]. Reference [55] gives a link for accessing the datafile containing the positions of the ZFBS-Morph overlaps and the hexameric ZFP57 binding site in the build hg19.

### Creating plots of the density of ZFBS-morph overlaps in genomic DNA

With another Perl script, I obtained the genomic positions of DNA segments that covered 2 or more closely spaced ZFBS-Morph overlaps along the chromosomal DNA sequences. That script opened the file containing the positions of ZFBS-Morph overlaps for a specified chromosome. Subsequently, the script scanned the file to count and to report the number of ZFBS-Morph overlaps within an 850-base window. I chose the window-size by trial and error [14]. Windows covering less than 850 bases tended to give superfluous spikes. Larger widows tended to produce false-peaks. By ignoring their isolated occurrences, the script removed background noise. Next, I combined and tailored the outputs of the program for display as a custom track at the UCSC genome browser. In exploratory studies, I found that density peaks corresponding to 3 or more ZFBS-Morph overlaps appeared reliable. Peaks covering 2 overlaps could be true or false-positive [57]. Reference [56] gives a link for accessing the datafile of density-plots. For an overview about how to use the UCSC genome browser, see [81, 82].

## Supplementary information

**Additional file 1 Figure S1**. A candidate ICR for *PRDM8*. Large scale experimental studies listed *PRDM8* as acandidate imprinted gene [15]. Due to its vicinity to a robust density peak, my strategy also predicts that *PRDM8* is potential imprinted gene. **Figure S2.** A candidate ICR for *PCNT*. Large scale experimental studies listed *PCNT* as a candidate imprinted gene [15]. Since *PCNT* includes 2 intragenic robust density peaks, my strategy predicts that *PCNT* is a potential imprinted gene. **Figure S3**. A density peak in the vicinity of *WDR60*. Results of large-scale experimental studies listed *WDR60* as a candidate imprinted gene [15]. within *WDR60*, I noticed a peak covering 2 ZFBS-morph overlaps. This peak could be a false or a true-positive. If false-positive, then another peak -far upstream of (*WDR60*)- could be a candidate ICR for regulating parent-of-origin specific expression of both *WDR60* and *ESYT2*. The latter gene encodes a protein (synaptotagmin-like protein 2) that belongs to a family of membranous Ca2 + −sensors. **Figure S4**. The positions of density peaks with respect to the ICR of *H19* – *IGF2* imprinted domain. In a few cases, an ICR encompasses two density peaks. The figure below includes a track displaying “Updated CTCF Binding sites predictions”. See references [25, 26] for updated positions of the unique repeats and CTCF sites upstream of *H19* TSS. Results of the ENCODE ChIPs do not support the existence of the predicted CTCF site 5 described previously [24]. Furthermore, in results of ChIPs displayed at the UCSC genome browser, I noticed a chromatin boundary consisting of CTCF, RAD21, and SMC3 in a CpG island upstream of *H19* TSS [25]. I named the predicted site in that island CTCF site 8. **Figure S5**. A peak in the density plots correctly locating the KvDMR in the *KCNQ1* imprinted domain. **Figure S6**. A candidate ICR for a potential imprinted gene (*CITED2*). This gene encodes a regulator of transcription. Absence of *Cited2* in mouse embryos caused congenital heart disease by perturbing left-right patterning of the body axis [31]. Deleterious mutations in *CITED2* cause VSD2 –ventricular septal defect 2 [75]. **Figure S7**. A candidate ICR for a potential imprinted gene (*FUCA2*). Note the position of the candidate ICR with respect to the SNP (rs72992630) and primers used to deduce that *FUCA2* is biallelically expressed gene [19]. The SNP and primers are not in the vicinity of the 1st exon of the transcript that is associated with a candidate ICR. **Figure S8**. A candidate ICR for a potential imprinted gene (*SAMD5*). A study found that *SAMD5* was overexpressed in prostate cancer and had powerful prognostic ability for predicting postoperative biochemical recurrence after radical prostatectomy [33]. **Figure S9**. A density peak predicted a candidate ICR for a potential imprinted gene (*ZBTB2*). In mouse embryonic stem cells, ZBTB2 dynamically interacted with nonmethylated CpG island promoters and regulated differentiation [35]. In colorectal cancer, the abnormal forms of *ZBTB2* increased cell proliferation [36]. **Figure S10**. Density peaks mapping to known parent-of-origin specific transcripts. One peak correctly located the ICR at the *MEST* locus. This ICR is intragenic and encompasses the TSSs of *MESTIT1* (a noncoding RNA gene) and a subset of *MEST* transcripts. *KLF14* is a known imprinted gene [30]. Density-plots predicted a candidate ICR regulating its imprinted expression. **Figure S11**. In density-plots, a peak correctly located the ICR regulating the expression of *INPP5_v2*.

## Data Availability

You can access the data via the following links. The positions of ZFBS and ZFBS-Morph overlaps in the build hg19 of the human genome: https://purr.purdue.edu/publications/3208/1 Density of ZFBS-Morph overlaps in the build hg19 of the human genome: https://purr.purdue.edu/publications/2967/1 The positions of the MLL morphemes in the build hg19 of the human genome: https://purr.purdue.edu/publications/1639/1

## Abbreviations

CGIs: CpG islands
