# Supplementary material for: Discovering candidate imprinted genes and imprinting control regions in the human genome
Source: BMC Genomics. 2020 May 31;21:378. doi: 10.1186/s12864-020-6688-8 (PMC7262774; doi:10.1186/s12864-020-6688-8)

Supplemental Figures

Fig. S1. A candidate ICR for *PRDM8*. Large scale experimental studies listed *PRDM8* as a candidate imprinted gene [15]. Due to its vicinity to a robust density peak, my strategy also predicts that *PRDM8* is potential imprinted gene.

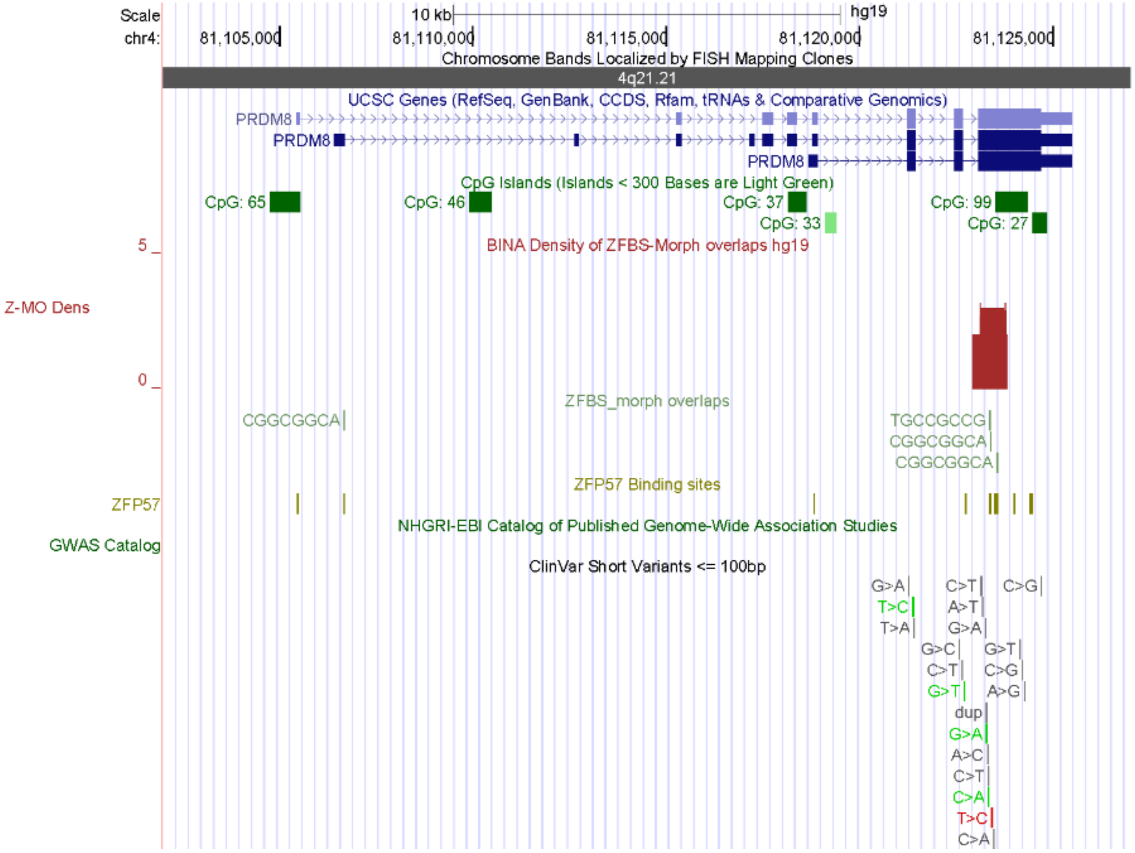

Next page

Fig. S2. A candidate ICR for *PCNT*. Large scale experimental studies listed *PCNT* as a candidate imprinted gene [15]. Since *PCNT* includes 2 intragenic robust density peaks, my strategy predicts that *PCNT* is a potential imprinted gene.

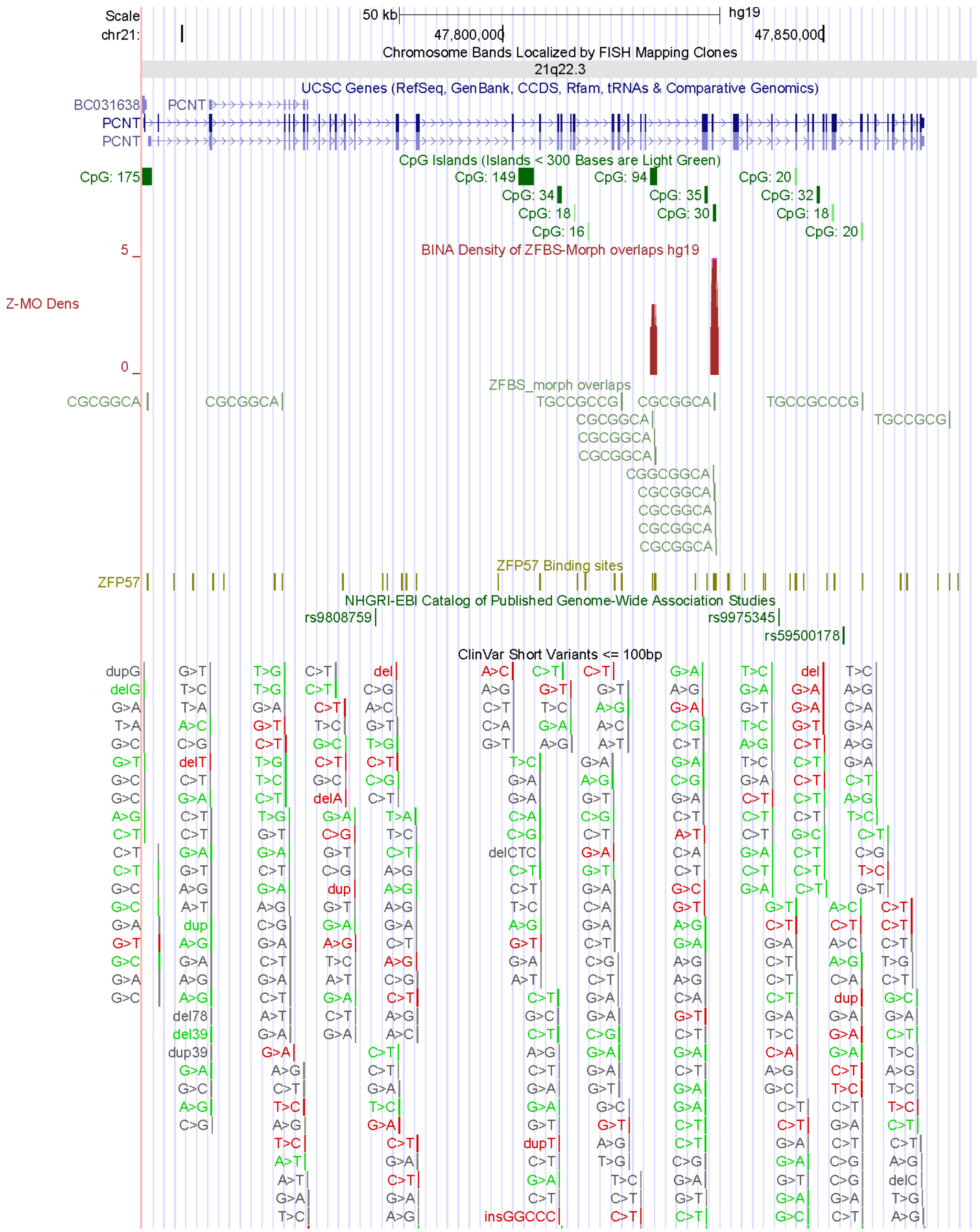

Fig. S3. A density peak in the vicinity of *WDR60*. Results of large-scale experimental studies listed *WDR60* as a candidate imprinted gene [15]. within *WDR60*, I noticed a peak covering 2 ZFBS-morph overlaps. This peak could be a false or a true-positive. If false-positive, then another peak -far upstream of (*WDR60*)- could be a candidate ICR for regulating parent-of-origin specific expression of both *WDR60* and *ESYT2*. The latter gene encodes a protein (synaptotagmin-like protein 2) that belongs to a family of membranous Ca2^{+}-sensors .

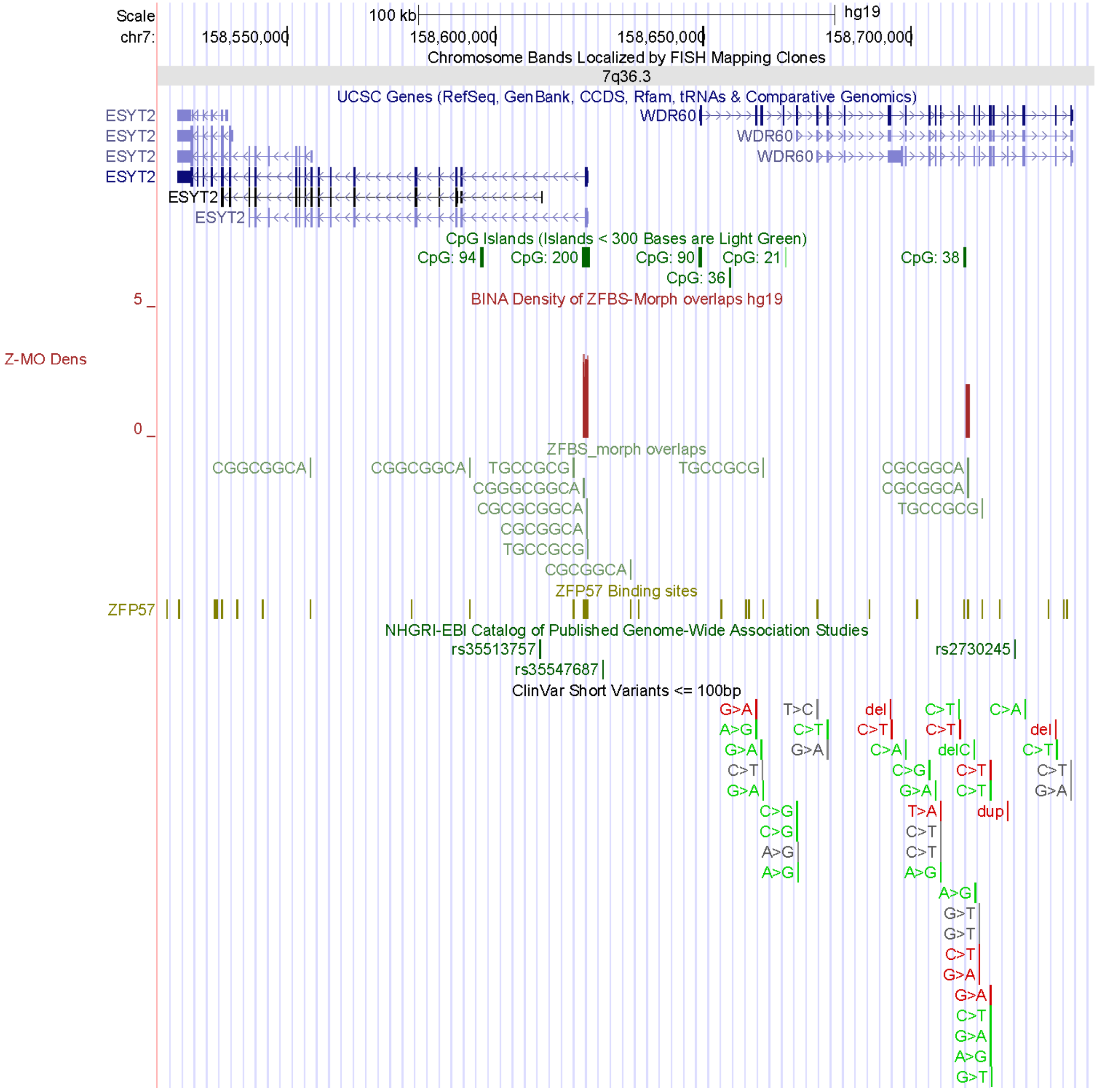

Fig. S4. The positions of density peaks with respect to the ICR of *H19 – IGF2* imprinted domain. In a few cases, an ICR encompasses two density peaks. The figure below includes a track displaying "Updated CTCF Binding sites predictions". See references [25, 26] for updated positions of the unique repeats and CTCF sites upstream of *H19* TSS. Results of the ENCODE ChIPs do not support the existence of the predicted CTCF site 5 described previously [24]. Furthermore, in results of ChIPs displayed at the UCSC genome browser, I noticed a chromatin boundary consisting of CTCF, RAD21, and SMC3 in a CpG island upstream of *H19* TSS [25]. I named the predicted site in that island CTCF site 8.

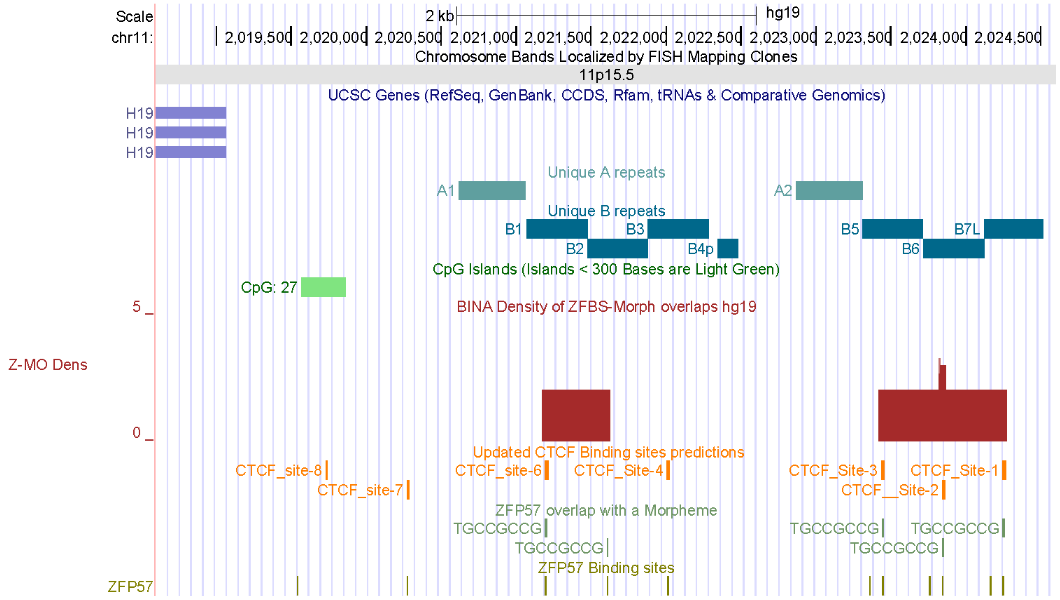

Fig. S5. A peak in the density plots correctly locating the KvDMR in the *KCNQ1* imprinted domain.

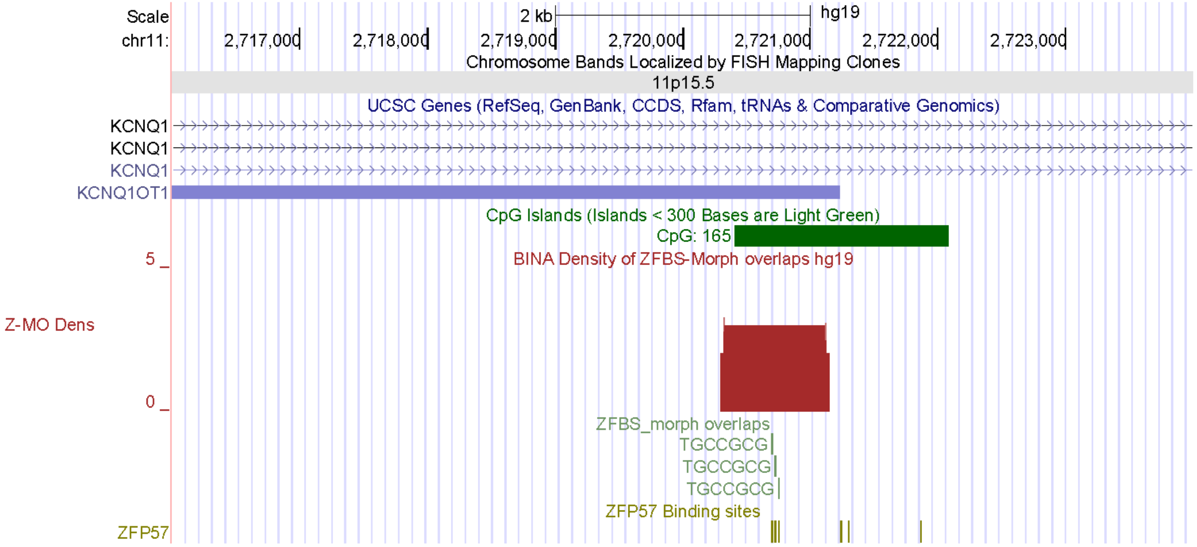

Fig. S6. A candidate ICR for a potential imprinted gene (*CITED2*). This gene encodes a regulator of transcription. Absence of *Cited2* in mouse embryos caused congenital heart disease by perturbing left-right patterning of the body axis [31]. Deleterious mutations in *CITED2* cause VSD2 –ventricular septal defect 2 [75].

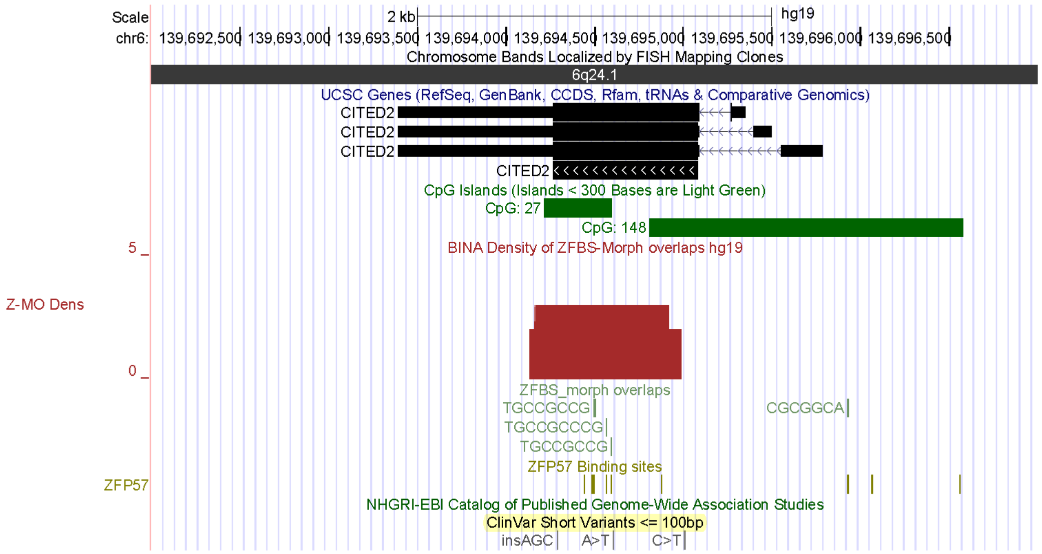

Fig. S7. A candidate ICR for a potential imprinted gene (*FUCA2*). Note the position of the candidate ICR with respect to the SNP (rs72992630) and primers used to deduce that *FUCA2* is biallelically expressed gene [19]. The SNP and primers are not in the vicinity of the 1st exon of the transcript that is associated with a candidate ICR.

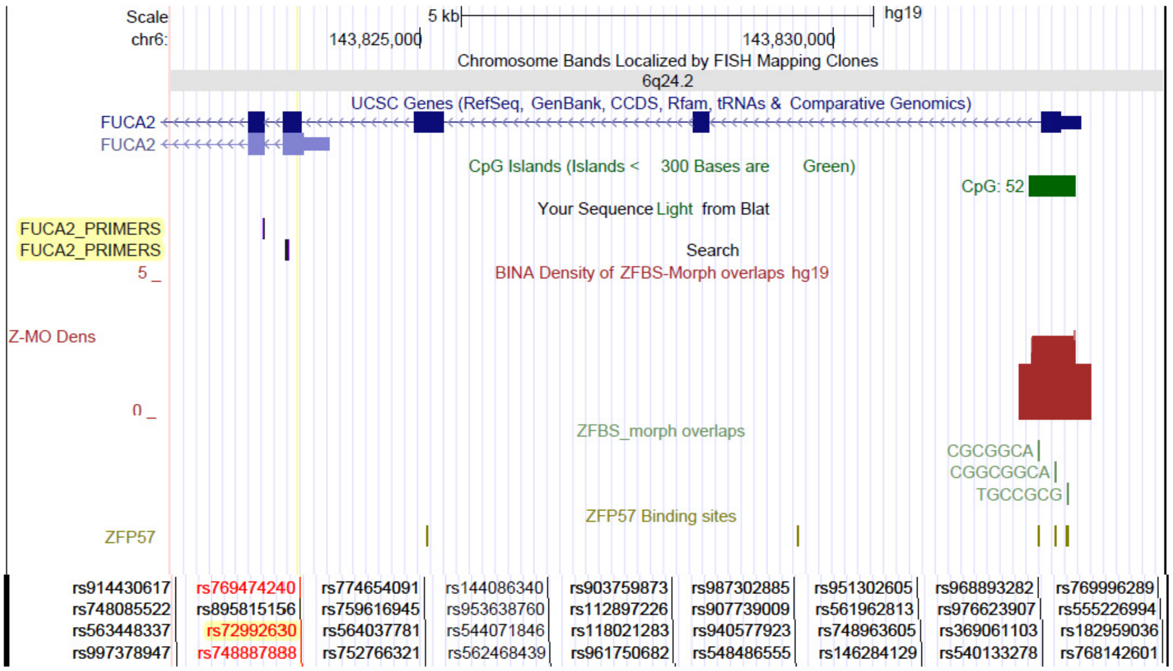

Fig. S8. A candidate ICR for a potential imprinted gene (*SAMD5*). A study found that *SAMD5* was overexpressed in prostate cancer and had powerful prognostic ability for predicting post-operative biochemical recurrence after radical prostatectomy [33].

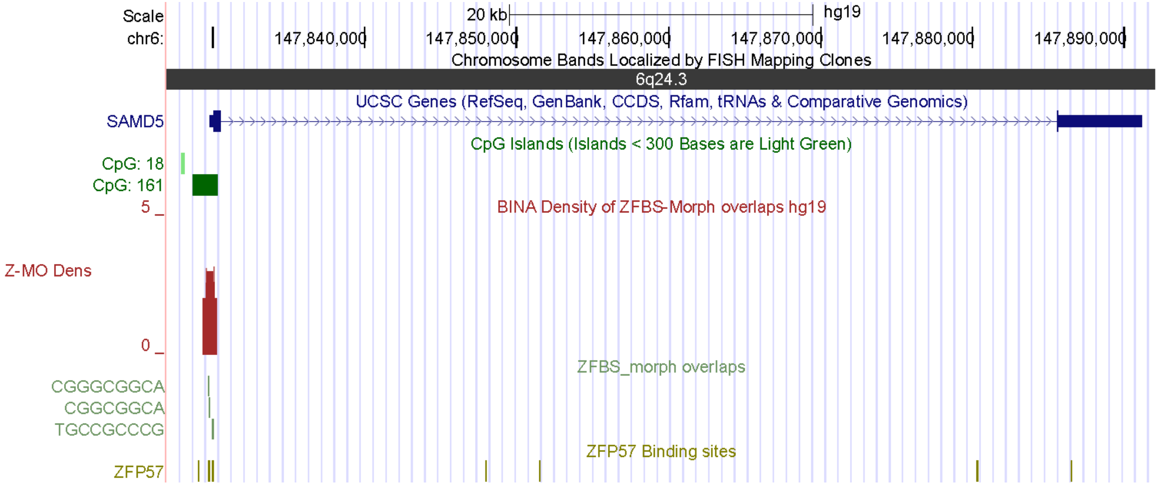

Fig. S9. A density peak predicted a candidate ICR for a potential imprinted gene (*ZBTB2*). In mouse embryonic stem cells, ZBTB2 dynamically interacted with nonmethylated CpG island promoters and regulated differentiation [35]. In colorectal cancer, the abnormal forms of *ZBTB2* increased cell proliferation [36].

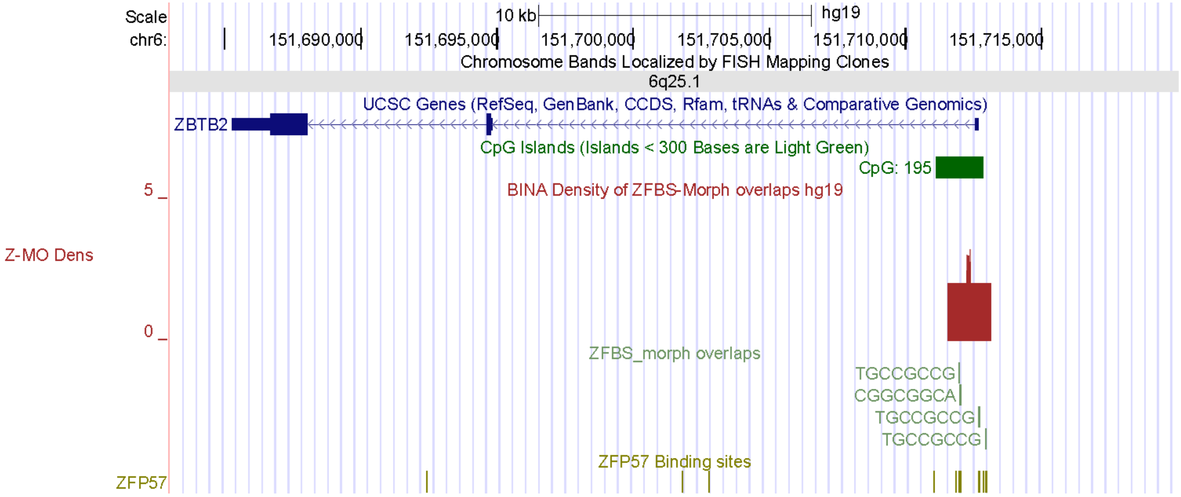

Fig. S10. Density peaks mapping to known parent-of-origin specific transcripts. One peak correctly located the ICR at the *MEST* locus. This ICR is intragenic and encompasses the TSSs of *MESTIT1* (a noncoding RNA gene) and a subset of *MEST* transcripts. *KLF14* is a known imprinted gene [30]. Density-plots predicted a candidate ICR regulating its imprinted expression.

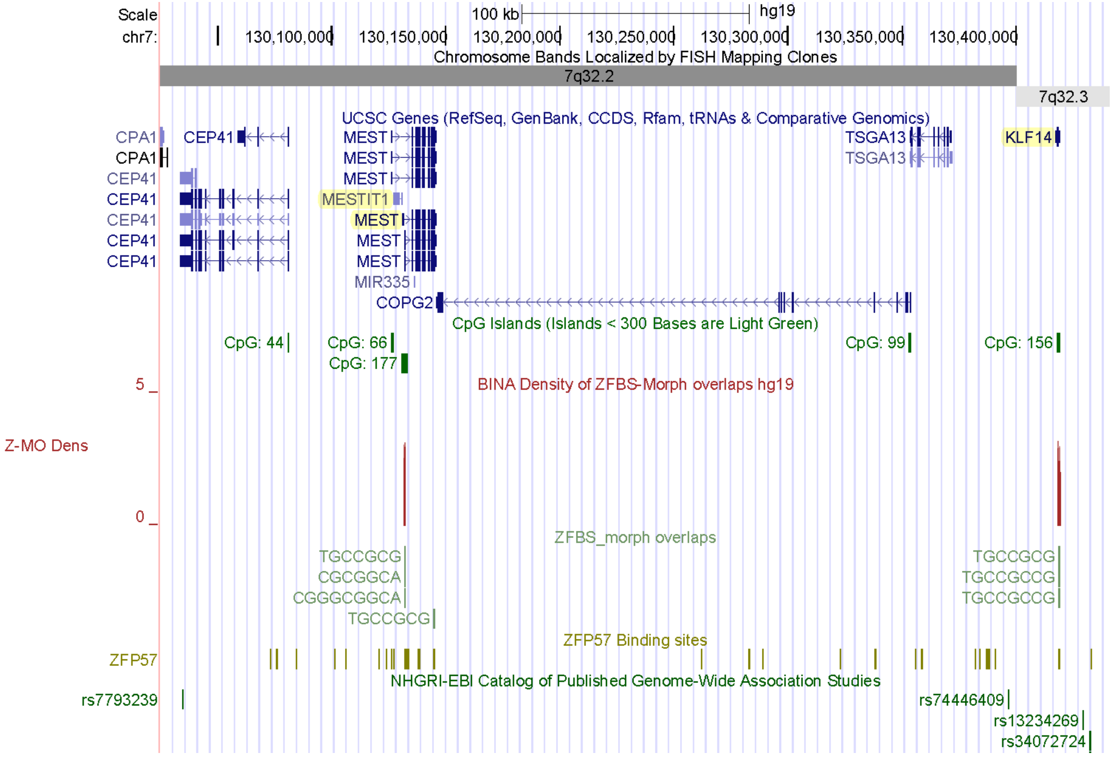

Fig. S11. In density-plots, a peak correctly located the ICR regulating the expression of *INPP5_v2*.

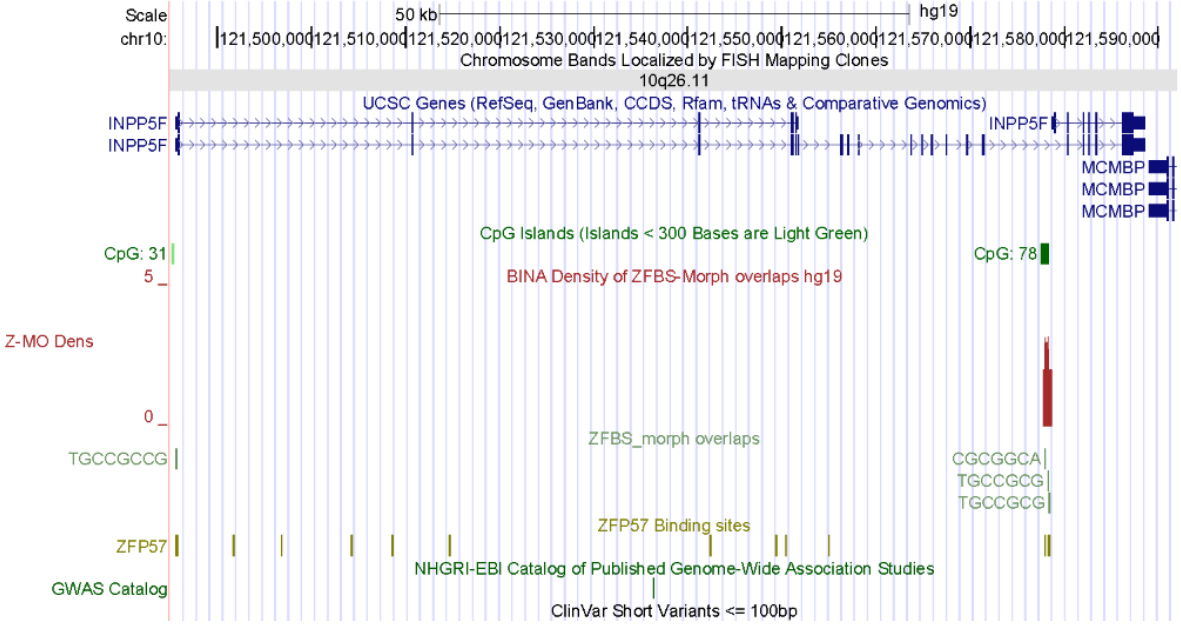

Supplement: Supplementary file 1 — Additional file 1 Figure S1. A candidate ICR for PRDM8. Large scale experimental studies listed PRDM8 as acandidate imprinted gene [15]. Due to its vicinity to a robust density peak, my strategy also predicts that PRDM8 is potential imprinted gene. Figure S2. A candidate ICR for PCNT. Large scale experimental studies listed PCNT as a candidate imprinted gene [15]. Since PCNT includes 2 intragenic robust density peaks, my strategy predicts that PCNT is a potential imprinted gene. Figure S3. A density peak in the vicinity of WDR60. Results of large-scale experimental studies listed WDR60 as a candidate imprinted gene [15]. within WDR60, I noticed a peak covering 2 ZFBS-morph overlaps. This peak could be a false or a true-positive. If false-positive, then another peak -far upstream of (WDR60)- could be a candidate ICR for regulating parent-of-origin specific expression of both WDR60 and ESYT2. The latter gene encodes a protein (synaptotagmin-like protein 2) that belongs to a family of membranous Ca2 + −sensors. Figure S4. The positions of density peaks with respect to the ICR of H19 – IGF2 imprinted domain. In a few cases, an ICR encompasses two density peaks. The figure below includes a track displaying “Updated CTCF Binding sites predictions”. See references [25, 26] for updated positions of the unique repeats and CTCF sites upstream of H19 TSS. Results of the ENCODE ChIPs do not support the existence of the predicted CTCF site 5 described previously [24]. Furthermore, in results of ChIPs displayed at the UCSC genome browser, I noticed a chromatin boundary consisting of CTCF, RAD21, and SMC3 in a CpG island upstream of H19 TSS [25]. I named the predicted site in that island CTCF site 8. Figure S5. A peak in the density plots correctly locating the KvDMR in the KCNQ1 imprinted domain. Figure S6. A candidate ICR for a potential imprinted gene (CITED2). This gene encodes a regulator of transcription. Absence of Cited2 in mouse embryos caused congenital heart disease by [file 12864_2020_6688_MOESM1_ESM.pdf]
